# Design, Synthesis and In Vitro Biological Activity of Novel C-7 Methylene Congeners of Furanoallocolchicinoids

**DOI:** 10.3390/pharmaceutics15041034

**Published:** 2023-03-23

**Authors:** Iuliia A. Gracheva, Elena V. Svirshchevskaya, Ekaterina S. Shchegravina, Yulia B. Malysheva, Alsu R. Sitdikova, Alexey Yu. Fedorov

**Affiliations:** 1 Department of Organic Chemistry, Nizhny Novgorod State University, Gagarina Av. 23, Nizhny Novgorod 603950, Russia; 2 Department of Immunology, Shemyakin-Ovchinnikov Institute of Bioorganic Chemistry RAS, Miklukho-Maklaya 16/10, Moscow 117997, Russia

**Keywords:** colchicine, antiproliferative activity, tubulin, activated C=C bond

## Abstract

A series of novel heterocyclic colchicine derivatives bearing a C-7 methylene fragment were synthesized via Wittig, Horner–Wadsworth–Emmons and Nenajdenko–Shastin olefination approaches. The in vitro biological activities of the most promising compounds were investigated using MTT assays and cell cycle analyses. Compounds with an electron withdrawing group on the methylene fragment exhibited substantial antiproliferative activity towards COLO-357, BxPC-3, HaCaT, PANC-1 and A549 cell lines. The spatial orientation of the substituent at the double bond significantly influenced its biological activity.

## 1. Introduction

**Figure 1:**
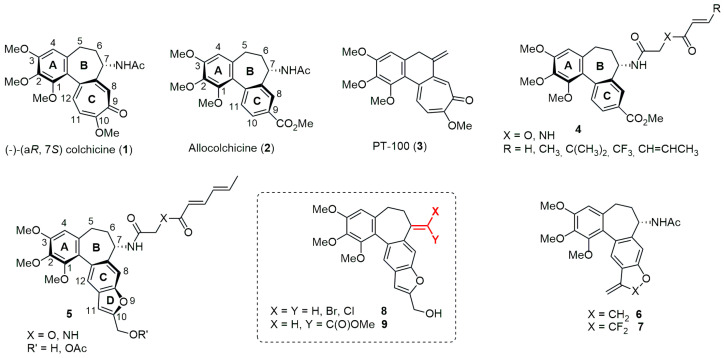
Colchicine (**1**), allocolchicine (**2**) and related compounds with olefinic fragments.

Colchicine (**1**) is a naturally occurring alkaloid derived from *Colchicum*, *Merendera* or *Gloriosa* plants. Colchicine is currently used to treat acute gout, Behçet’s disease, Mediterranean fever, chondrocalcinosis, systemic scleroderma and amyloidosis [1,2]. Due to its strong anti-inflammatory activity, colchicine [3,4] has been considered for treating numerous inflammatory diseases of different origins, including COVID-19 [5,6,7]. However, the high systemic toxicity of colchicine significantly restricts its clinical application [8]. During recent decades, numerous attempts to change colchicine’s structure have been undertaken, aiming to reduce its general toxicity while maintaining its antiproliferative activity [9]. To date, a plethora of new derivatives of colchicine have been synthesized and tested in vitro and in vivo [9]. A number of selected promising compounds are presented in Figure 1. Most of them contain a double carbon–carbon bond that plays an important role as a pharmacophore.

The approach based on the contraction of ring *C* and formation of heterocycle fragment *D* led to several promising compounds exhibiting antiproliferative activity in the low nanomolar range. Thus, allocolchicine **2** can be considered as the parent structure for the compounds **4** and **5** with Michael acceptor fragments in their side chains, as well as for highly active molecules **6** and **7** that exhibit a six-fold decreased acute toxicity in comparison with colchicine [10,11]. Another impressive example of colchicine structure modification was demonstrated by Prof. H.-G. Schmalz’s research group. They synthesized colchicinoid PT-100 (**3**), which demonstrated a strong synergetic pro-apoptotic effect in combination with vincristine on resistant cell lines [12]. Based on previous research, we suggest novel colchicinoids **8** and **9**, bearing activated double bonds in the pseudo-benzylic position in ring *B*. This C=C bond can possibly interact with thiol groups in cysteine residues, increasing the efficiency and overcoming multidrug resistance.

## 2. Materials and Methods

### 2.1. General Information

^1^H NMR and ^13^C NMR spectra were recorded in DMSO-*d*_6_ at 25 °C on an Agilent DDR2 400 spectrometer with operating frequencies of 400 MHz for ^1^H and 101 MHz for ^13^C. Chemical shifts (δ) are reported in parts per million (ppm) from TMS using the residual solvent resonance (DMSO-*d*_6_: 2.50 ppm for ^1^H NMR, 39.52 ppm for ^13^C NMR). Signal assignments in the proton spectra were based on comparison [13,14]. Mass spectra were recorded on a DSQ mass spectrometer with a quadrupole mass analyzer. The temperature of the ion source was 230 °C and ionization was carried out by electrons with an energy of 70 eV. Elemental analyses were performed on an Elementar (Vario Micro Cube) instrument. Column chromatography was performed using Merck Kieselgel 60 (70–230 mesh). Commercially available reagents (Aldrich, AlfaAesar and Acros) were used without additional purification. Solvents were purified according to the standard procedures. The petroleum ether (PE) used corresponds to the 40–70 °C fraction.

### 2.2. Biological Assays

#### 2.2.1. Cell Cultures

Murine lymphocytes EL-4, human hepatocytes HepG2, pancreatic adenocarcinoma COLO357, pseudonormal keratinocytes HaCaT, human adenocarcinoma BxPC-3, pancreatic carcinoma PANC-1 and adenocarcinomic alveolar epithelial cells A549 (IBCh collection, Moscow, Russia) were grown in RPMI-1640 or DMEM media supplemented with 10% fetal calf serum (FCS) and pen-strep-glut (complete culture medium) (all from PanEco, Moscow, Russia) in a CO_2_ incubator at 37 °C. Cells were passaged using a Trypsin/EDTA solution (PanEco, Moscow, Russia) twice a week.

BALB/c mice were euthanized by cervical dislocation. Spleens were collected and homogenized in saline. Red blood cells were lysed by 0.83% NH_4_Cl solution, washed twice with saline, transferred to the complete culture medium and stimulated at 5 × 10^6^/mL of cells with concanavalin A (5 µg/mL) for 72 h. Activated splenocytes were washed in saline, transferred to culture medium and used for the analyses.

#### 2.2.2. MTT Assay

The antiproliferative effect of the compounds was estimated by a standard 3-(4,5-dimethyl-2-thiazolyl)-2,5-diphenyl-2H-tetrazolium bromide (MTT, Sigma, St. Louis, MO, USA) test. To do this, the cells were transferred into a flat-bottomed 96-well plate, 5 × 10^3^ per well, in which the preparations were titrated in advance. The plates were incubated in a CO_2_ incubator for 72 h, and 250 µg·mL^−1^ MTT was added for the last 4 h. After incubation, the culture medium was removed and 100 μL of DMSO was added to each well to dissolve the formazan. The plates were analyzed on a plate spectrophotometer at a wavelength of 540 nm. The inhibition index (II) was calculated by the formula II = 1 − OD_experiment_/OD_control_, where OD is the optical density of the solution. The IC_50_ values were determined as 50% inhibition index.

#### 2.2.3. Confocal Microscopy

K562 cells, 10^5^, were seeded onto sterile cover glasses (10^5^/per well) and incubated in a CO_2_ incubator at 37 °C until the cells adhered. Preparation **9Z** was added at 500 nM concentration and cells were incubated for 18 h at 37 °C. At the end of the incubation, the cells were fixed with 1% PFA and washed three times with PBS 0.01% Triton X100. The fixed and permeabilized cells were stained with anti-murine β-tubulin antibody (Santa Cruze, CA, USA), phalloidin AlexaFluor 488 (Applied Biosystems, Foster City, CA, USA) and nuclear dye Hoechst 33342 (Merck KGaA, Darmstadt, Germany) for 1 h. Secondary anti-murine IgG-AlexaFluor555 (Merck KGaA, Darmstadt, Germany) was used to vizualize β-tubulin. Cells were polymerized by Mowiol (Calbiochem, Nottingham, UK) and analyzed using a TE 2000 Eclipse confocal microscope (Nikon, Tokyo, Japan).

#### 2.2.4. Cell Cycle Analysis

The cell cycle was analyzed using PI-stained DNA. Cells from colchicinoid-treated cultures were collected, washed in ice-cold PBS, fixed by the addition of 70% ethanol and left for 2 h at −20 °C. Thereafter, the cells were washed twice in PBS, stained with 50 μg/mL of propidium iodide (Merck KGaA, Darmstadt, Germany) in PBS, treated with 10 µg/mL of RNAse and analyzed by flow cytometry using an FACScan device (BD, Franklin Lakes, NJ, USA). A total of 2000 events were collected. The results were analyzed using FlowJo 10 software (BD, Franklin Lakes, NJ, USA).

## 3. Results

### 3.1. Chemistry

**Scheme 1:**
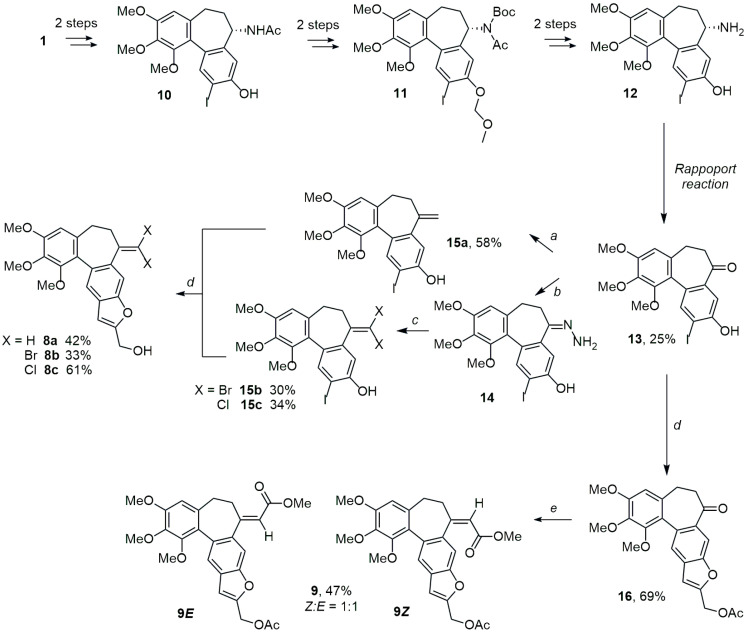
Synthesis of colchicinoids **8** and **9**. Reagents and conditions: (a) Ph_3_PMeI, *t*-BuOK, THF, rt, 2 h; (b) NH_2_-NH_2_·H_2_O, EtOH, 80 °C, 3.5 h; (c) CX_4_ (**11b** X = Br, **11c** X = Cl), H_2_NCH_2_CH_2_NH_2_, CuCl (10%), EtOH, rt, 24 h; (d) propargylic alcohol for **8a**–**c** and propargyl acetate for **9**, Pd(OAc)_2_ (0.05 equiv.), CuI (0.1 equiv.), AcOK (3 equiv.), Ph_3_P (0.15 equiv.), MeCN, 70 °C 6–8 h; (e) (CH_3_O)_2_P(O)CH_2_CO_2_CH_3_, NaH, THF, 0 °C–rt–65 °C, 20 h.

The synthesis of target furano-allocolchicinoids **8** and **9** bearing a methylene moiety at the C-7 position began with the preparation of ketone **13** in a seven-step reaction sequence, according to a published procedure [14]. Briefly, colchicine **1** was converted into iodo-colchinol **10** under Windaus conditions [15]; then, two acid-labile protecting groups (MOM and Boc) were introduced to facilitate the deacetylation process of colchicinoid **11**. After acid-catalyzed cleavage of both protecting groups, N-deacetyliodocolchinol **12** was obtained. The above-mentioned amine was converted into ketone **13** by the Rapoport reaction [16] (total yield: 25%). Olefination of compound **13** under Wittig and Nenajdenko–Shastin [17] reaction conditions gave methylidene **15a** and dibromo- (**15b**) and dichloro-substituted (**15c**) derivatives, respectively (Scheme 1). In the latter cases of compounds **15b** and **15c**, ketone **13** was first converted into hydrazone **14** by reaction with hydrazine.

The transformation of hydrazine **14** into corresponding alkenes **15b** and **15c** was performed using CX_4_ (X = Cl or Br) and a catalytic amount of copper(I) salt. The reaction began with the oxidation of Cu(I) to Cu(II), which, in turn, oxidized the hydrazone **14** to diazoalkane. After the decomposition of the latter, a Cu-carbeniod was formed as the key intermediate. In the last step, this was treated with CX_4_, resulting in the formation of alkene **15b** or **15c**. Finally, a furan ring D was formed via a domino Sonogashira/cyclization cross-coupling reaction of colchicinoids **15a**–**c** with the corresponding alkynes, which has been described earlier [13]. Target furano-allocolchicinoids **8a**–**c** were obtained with moderate (33–61%) yields (Scheme 1).

For the synthesis of derivative **9**, ketone **13** was converted into furane derivative **16**. The latter was treated with methyl 2-(dimethoxyphosphoryl)acetate and sodium hydride, resulting in the formation of alkene **9** (Z/E ratio 1:1). For details concerning the assignment of E- and Z-configurations for compounds **9E** and **9Z**, respectively, see the Supporting Information (2D NMR spectra).

### 3.2. In Vitro Bioassays

All final compounds were tested on a number of cancer cell lines (COLO-357, BxPC-3, HaCaT, PANC-1 and A549). The antiproliferative activity was measured using the standard MTT assay. The results are summarised in Table 1.

The results indicated that the antiproliferative activity of the target compounds depends on the substituent on the C=C bond. Unsubstituted alkene **8a** demonstrated no activity, whereas the presence of electron withdrawing groups, in particular, an ester group, increases the potency of the compound. However, the geometry of the conjugated ester in compound **9** also influences the in vitro activity. Due to the significant difference in IC_50_ of compounds **9Z** and **9E**, we investigated the in vitro effects of **9E** in more detail.

**Figure 2:**
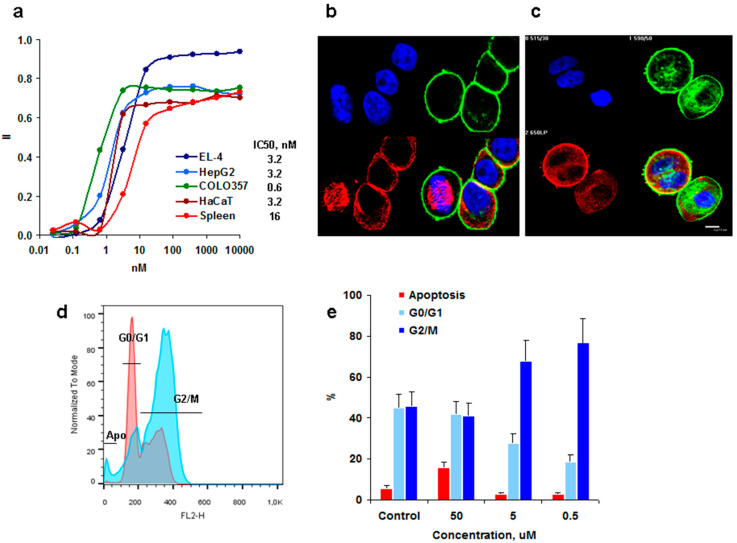
Functional characteristics of **9E**: (**a**) Antiproliferative activity of **9E** against EL-4, HepG2, COLO357 and HaCaT cell lines and against normal mitogen activated splenocytes (spleen); the IC_50_ of the corresponding cells is shown on the right side. SD were around 5–15%, and are not shown for a better visualization. (**b**,**c**) Confocal microscopy analysis of β-tubulin (red) and F-actin (green) staining of K562 cells without (**b**) and with 500 nM of **9E**. (**c**) Nuclei are stained with Hoechst 33342 (blue). Scale bar 15 µm. (**d**,**e**) Cell cycle analysis of K562 cells in control ((**d**), pink histogram) and after 48 h incubation with 500 nM of **9E** ((**d**), blue histogram) and dose-dependent distribution of cell cycle phases. G2/M—cells with doubled DNA; G0/G1—nonproliferating cells with single DNA; Apo—apoptotic cells.

The antiproliferative activity of **9E** was investigated using various cell lines (Figure 2a). Cell lines originating from normal tissue, such as keratinocyte HaCaT or embryo kidney HEK293 cells, are often used as normal controls. However, telomerase activities in all immortalized cells either from cancer or normal tissues make them behave similar to cancer cells. To compare the compound’s toxicity towards immortalized and normal cells, we used mitogen-activated murine splenocytes. Activation of lymphocytes with mitogen concanavalin A stimulates their proliferation. It appeared that this compound exhibited 5 times less toxicity against the activated splenocytes in comparison with immortalized cell lines (COLO357, EL-4, HaCaT). Additionally, **9E** induces cell cycle arrest, a significant accumulation of cells in G2/M and a restriction of cell population in G0/G1 (Figure 2d,e). In general, the biological in vitro effects of **9E** and colchicine **1** share much in common, but colchicinoid **9E** can potentially covalently bind with tubulin due the presence of the conjugated ester fragment that can act as a Michael acceptor.

## 4. Conclusions

The design, synthesis and in vitro biological evaluation of novel colchicinoids bearing an activated olefin fragment on ring *B* are presented. The synthetic protocol described in this paper allows the introduction of olefin fragments with various substituents, with the aim to control the compound’s activity. Compound **9E**, bearing the conjugated ester moiety, exhibits low nanomolar antiproliferative activity and moderate selectivity towards selected cancer cell lines. It induces cell cycle arrest and cell accumulation in the G2/M stage. In contrast, compound **9Z** demonstrates a reduced antiproliferative activity in comparison with its isomer **9E**, which clearly indicates the importance of the spatial orientation of the ester group on the olefin fragment. The ability to covalently bond with the target protein and other details of the ligand–protein interaction require further investigation.

## 5. Experimental Section

### Synthetic Procedures

Synthesis of intermediates **10**–**13** and **16** were performed according to a literature protocol described in [14].

Synthesis of 2-iodo-9,10,11-trimethoxy-5-methylene-6,7-dihydro-5H-dibenzo[a,c]cycloheptene-3-ol **15a**

Anhydrous THF (2 mL) was added to a flask containing Ph_3_PMeI (286.2 mg, 0.708 mmol) under an argon atmosphere. *t*-BuOK (79.4 mg, 0.708 mmol) was added and the mixture was vigorously stirred for 45 min until a stable bright yellow color appeared. Then, ketone **13** was added and the mixture was stirred for 2 h. After the completion of the reaction, the precipitate was filtered through a Schott filter and washed with n-pentane. The solution was evaporated under reduced pressure. The residue was purified by column chromatography on silica gel with the eluent PE–EtOAc–EtOH (5:1:1) to afford **15a** (89.2 mg, 58%) as white crystals, m.p. 125–127 °C.

^1^H NMR (400 MHz, DMSO-*d*_6_, δ, ppm, *J*/Hz): 10.49 (s, 1H, OH), 7.60 (s, 1H, H(11)), 6.73 (s, 1H, H(4)), 6.74 (s, 1H, H(8)), 4.92 (s, 1H, =CH_2_), 4.77 (s, 1H, =CH_2_), 3.79 (s, 3H, OCH_3_(1)), 3.72 (s, 3H, OCH_3_(2)), 3.42 (s, 3H, OCH_3_(3)), 2.88 (d, *J* = 15.2 Hz, 1H, CH_2_(5)), 2.69 (dd, *J* = 20.0, 9.1 Hz, 1H, CH_2_(5)), 2.47–2.33 (m, 2H, CH_2_(6)).

^13^C NMR (101 MHz, DMSO-*d*_6_, δ, ppm): 155.7, 152.0, 150.5, 148.3, 143.1, 140.5, 139.8, 135.60, 126.9, 124.1, 114.4, 113.9, 108.1, 82.8, 60.6, 60.4, 55.9, 40.9, 31.1.

Elemental analysis: found (%): C, 52.12; H, 4.41. C_19_H_19_IO_4_. Calcd. (%): C, 52.07; H, 4.37.

MS (EI): *m*/*z* (%) = 438 (100), 280 (14), 181 (10), 152 (11).

Synthesis of 5-hydrazono-2-iodo-9,10,11-trimethoxy-6,7-dihydro-5H-dibenzo[a,c]-[7]annulen-3-ol **14**

To a solution of ketone **13** (300.0 mg, 0.682 mmol) in 10.0 mL of EtOH, NH_2_-NH_2_·H_2_O (100 μL, 2.044 mmol) was added under argon. The resulting mixture was refluxed for 3.5 h. After the completion of the reaction, the solution was cooled to room temperature. Pure product **14** in the form of white crystals (147.0 mg, 47%) was isolated by silica gel column chromatography with the eluent PE–EtOAc–EtOH (5:1:1), m.p. 133–135 °C.

^1^H NMR (400 MHz, DMSO-*d*_6_, δ, ppm, *J*/Hz): 10.39 (s, 1H, OH), 7.61 (s, 1H, H(11)), 6.90 (s, 1H, H(8)), 6.77 (s, 1H, H(4)), 6.18 (s, 2H, NH_2_), 3.81 (s, 3H, OCH_3_(1)), 3.73 (s, 3H, OCH_3_(2)), 3.47 (s, 3H, OCH_3_(3)), 2.67–2.55 (m, 4H, CH_2_(5) и CH_2_(6)).

^13^C NMR (101 MHz, DMSO-*d*_6_, δ, ppm): 155.4, 152.1, 150.7, 147.3, 140.6, 140.3, 140.2, 136.6, 126.2, 123.4, 114.8, 107.5, 83.2, 60.5, 60.5, 55.8, 34.5, 29.6.

Elemental analysis: found (%): C, 47.55; H, 4.17. C_18_H_19_IN_2_O_4_. Calcd (%): C, 47.59; H, 4.22.

MS (EI): *m*/*z* (%) = 454 (100), 453 (45), 452 (19), 437 (16), 423 (8).

General procedure for the synthesis of compounds **15b**, **15c**

Freshly purified CuCl (10 mol %) and ethylenediamine (3.4 mL per 1 mmol of hydrazone) were added to a solution of freshly prepared hydrazone **14** in EtOH (49.0 mL per 1 mmol of hydrazone). After careful addition of 2 equiv. CX_4_ (X = Br, Cl) at 20 °C, the reaction mixture was stirred for 24 h at room temperature. At the end of the reaction, the mixture was neutralized with a saturated solution of NH_4_Cl (aq.). After evaporation of the solvent, the resulting solution was extracted three times with CH_2_Cl_2_. The organic layer was concentrated under reduced pressure. A mixture of petroleum ether, ethyl acetate and ethanol, or dichloromethane and methanol, was used as the eluent.

5-(Dibromomethylene)-2-iodo-9,10,11-trimethoxy-6,7-dihydro-5H-dibenzo[a,c]-[7]annulen-3-ol **15b**

Eluent: PE–EtOAc–EtOH (5:1:1), beige powder, 30% yield, m.p.167–169 °C.

^1^H NMR (400 MHz, DMSO-*d*_6_, δ, ppm, *J*/Hz): 10.35 (s, 1H, OH), 7.59 (s, 1H, H(11)), 7.05 (s, 1H, H(4)), 6.76 (s, 1H, H(8)), 3.81 (s, 3H, OCH_3_(1)), 3.76 (s, 3H, OCH_3_(2)), 3.49 (s, 3H, OCH_3_(3)), 2.46–2.35 (m, 2H, CH_2_(5)), 2.10–1.98 (m, 2H, CH_2_(6)).

^13^C NMR (101 MHz, DMSO-*d*_6_, δ, ppm): 155.7, 152.2, 150.0, 141.6, 140.5, 139.3, 135.0, 126.1, 122.7, 109.6, 109.5, 108.1, 81.7, 76.7, 64.4, 60.5, 60.5, 55.8, 29.4.

Elemental analysis: found (%): C, 38.25; H, 2.90. C_19_H_17_Br_2_IO_4_. Calcd (%): C, 38.29; H, 2.87.

MS (EI): *m*/*z* (%) = 596 (<1), 517 (2, -Br^79^), 515 (1, -Br^81^), 470 (100), 469 (37), 468 (24), 424 (61), 409 (36), 393 (31), 381 (18), 298 (33), 297 (38), 284 (45), 283 (68), 267 (60), 239 (48), 211 (41), 197 (52), 168 (50).

5-(Dichloromethylene)-2-iodo-9,10,11-trimethoxy-6,7-dihydro-5H-dibenzo[a,c]-[7]annulen-3-ol **15c**

Eluent: CH_2_Cl_2_–MeOH (100:1 → 50:1 → 20:1), beige powder, 34% yield, m.p. 171–173 °C.

^1^H NMR (400 MHz, DMSO-*d*_6_, δ, ppm, *J*/Hz): 10.35 (s, 1H, OH), 7.59 (s, 1H, H(11)), 7.05 (s, 1H, H(4)), 6.76 (s, 1H, H(8)), 3.81 (s, 3H, OCH_3_(1)), 3.76 (s, 3H, OCH_3_(2)), 3.49 (s, 3H, OCH_3_(3)), 2.47–2.33 (m, 3H, CH_2_(5), CH_2_(6)), 2.10–2.02 (m, 1H, CH_2_(6)).

^13^C NMR (101 MHz, DMSO-*d*_6_, δ, ppm): 155.7 (superposition of two signals), 152.2, 150.0, 141.6, 140.5, 139.3, 135.0, 126.1, 122.7, 109.6, 108.1, 81.7, 76.7, 64.4, 60.5, 60.5, 55.8, 29.4.

Elemental analysis: found (%): C, 45.00; H, 3.38. C_19_H_17_Cl_2_IO_4_. Calcd (%): C, 45.04; H, 3.35. MS (EI): *m*/*z* (%) = 470 (-H, -Cl, 16), 381 (8), 268 (37), 267 (50), 256 (63), 255 (84), 254 (89), 239 (66), 197 (67), 181 (63).

General procedure for the synthesis of compounds **8a**–**c**

Compound **15a**–**c** (1 equiv.), Pd(OAc)_2_ (5 mol.%), CuI (10 mol.%), PPh_3_ (15 mol.%) and KOAc (3 equiv.) were placed into a Schlenk flask under inert atmosphere and dry MeCN was added. Propargyl alcohol (1 equiv.) was then added dropwise. The resulting solution was stirred for 1 h at 60 °C, and then the temperature was raised to 80 °C and the solution was stirred for another 6–7 h. The progress of the reaction was monitored by TLC. After completion of the reaction, the solution was cooled to room temperature. The pure product was isolated by silica gel column chromatography. A mixture of petroleum ether, ethyl acetate and ethanol was used as the eluent.

1′,2′,3′-Trimethoxybenzo[5′,6′:5,4]-1H-1-methylene-6,7-dihydroxycyclohepta[3,2-f]-2″-hydroxy-methylbenzofuran **8a**

Eluent: PE–EtOAc–EtOH (6:1:1), white crystals, 42% yield, m.p. 112–114 °C.

^1^H NMR (400 MHz, DMSO-*d*_6_, δ, ppm, *J*/Hz): 7.54 (s, 1H, H(12)), 7.35 (s, 1H, H(8)), 6.78 (s, 1H, H(11)), 6.77 (s, 1H, H(4)), 5.48 (t, *J* = 5.8 Hz, 1H, OH), 4.98 (s, 1H, =CH_2_), 4.84 (s, 1H, =CH_2_), 4.58 (d, *J* = 5.8 Hz, 2H, -CH_2_OH), 3.82 (s, 3H, OMe), 3.75 (s, 3H, OMe), 3.33 (s, 3H, OMe), 2.96–2.89 (m, 1H, CH_2_(5)), 2.79–2.70 (m, 1H, CH_2_(6)), 2.56 (dd, *J* = 13.2, 3.1 Hz, 1H, CH_2_(6)), 2.41 (td, *J* = 13.2, 6.1 Hz, 1H, CH_2_(5)).

^13^C NMR (101 MHz, DMSO-*d*_6_, δ, ppm): 158.9, 153.4, 151.9, 150.7, 148.9, 140.5, 138.0, 135.39, 130.1, 128.9, 127.1, 125.5, 122.2, 122.0, 114.5, 109.5, 107.8, 103.3, 60.5, 60.1, 56.2, 55.7, 41.1, 31.0.

Elemental analysis: found (%): C, 72.12; H, 6.05. C_22_H_22_O_5_. Calcd (%): C, 72.16; H, 6.08.

MS (EI): *m*/*z* (%) = 366 (100), 335 (22), 304 (17), 292 (10), 205 (11), 189 (16), 165 (14), 94 (32).

1′,2′,3′-Trimethoxybenzo[5′,6′:5,4]-1H-1-dibromomethylene-6,7-dihydroxycyclohepta[3,2-f]-2″-hydroxymethylbenzofuran **8b**

Eluent: PE–EtOAc–EtOH (6:1:1), greenish crystals, 33% yield, m.p. 102–104 °C.

^1^H NMR (400 MHz, DMSO-*d*_6_, δ, ppm, *J*/Hz): 7.65 (s, 1H, H(12)), 7.56 (s, 1H, H(8)), 6.86 (br.s, 2H, H(11) and H(4)), 5.59 (br.s, 1H, OH), 4.61 (br.s, 2H, CH_2_OH), 3.84 (s, 3H, OCH_3_(1)), 3.76 (s, 3H, OCH_3_(2)), 3.39 (s, 3H, OCH_3_(3)), 3.06–2.99 (m, 2H, CH_2_(5)), 2.90–2.80 (m, 2H, CH_2_(6)).

^13^C NMR (101 MHz, DMSO-*d*_6_, δ, ppm): 161.4, 152.8, 152.6, 151.5, 140.9, 136.1, 135.8, 130.5, 128.1, 123.7, 123.3, 109.4, 107.6, 103.4, 60.5, 60.5, 56.3, 55.9, 47.8, 29.2.

Elemental analysis: found (%): C, 50.41; H, 3.85. C_22_H_20_Br_2_O_5_. Calcd (%): C, 50.37; H, 3.82.

1′,2′,3′-Trimethoxybenzo[5′,6′:5,4]-1H-1-dichloromethylene-6,7-dihydroxycyclohepta [3,2-f]-2″-hydroxymethylbenzofuran **8c**

Eluent: PE–EtOAc–EtOH (6:1:1), light beige powder, 61% yield, m.p. 120–122 °C.

^1^H NMR (400 MHz, DMSO-*d*_6_, δ, ppm, *J*/Hz): 7.55 (s, 1H, H(12)), 7.52 (s, 1H, H(8)), 6.79 (s, 1H, H(11)), 6.77 (s, 1H, H(4)), 5.45 (t, *J* = 5.9 Hz, 1H, OH), 4.58 (d, *J* = 5.8 Hz, 2H, CH_2_OH), 3.83 (s, 3H, OCH_3_(1)), 3.78 (s, 3H, OCH_3_(2)), 3.43 (s, 3H, OCH_3_(3)), 2.48–2.36 (m, 3H, CH_2_(5), CH_2_(6)), 1.78–1.72 (m, 1H, CH_2_(6)).

^13^C NMR (101 MHz, DMSO-*d*_6_, δ, ppm): 158.5, 153.8, 152.2, 150.2, 140.5, 136.6, 135.0, 128.3, 126.5, 124.2, 122.1, 108.0, 105.1, 103.3, 77.3, 64.5, 60.6, 60.4, 56.2, 55.8, 29.5, 15.4.

Elemental analysis: found (%): C, 60.70; H, 4.63. C_22_H_20_Cl_2_O_5_. Calcd (%): C, 60.73; H, 4.66.

MS (EI): *m*/*z* (%) = 435 (<1), 400 (-Cl^35^, 21), 399 (44), 398 (-Cl^37^, 68), 397 (100), 396 (58), 395 (43), 394 (28), 367 (18), 352 (54), 351 (33), 350 (26), 337 (18), 294 (11), 280 (11).

Synthesis of 1′,2′,3′-trimethoxybenzo[5′,6′:5,4]-1H-1-methoxycarbonylmethylene-6,7-dihydroxy-cyclohepta[3,2-f]-2″-hydroxymethylbenzofuran **9Z**, **9E**

To a stirred solution of NaH (60% in mineral oil, 16.9 mg, 0.422 mmol) in 3.0 mL of THF was added a solution of trimethylphosphinoacetate in 1.0 mL of THF at 0 °C under an inert atmosphere. The mixture was stirred at 0 °C for 1 h, then a solution of compound **16** in 1.2 mL of THF was carefully added. The resulting reaction mixture was stirred for 1.5 h at room temperature, and then at 60 °C for about 20 h. After evaporation of the solvent, pure product **9** in the form of Z and E isomers 1:1 (total 31.0 mg, 47%) was isolated as a white powder by column chromatography on silica gel with the eluent PE–EtOAc (3:1).

**9E**: m.p. 128–130 °C.

^1^H NMR (400 MHz, DMSO-*d*_6_, δ, ppm, *J*/Hz): 7.62 (s, 1H, H(12)), 7.50 (s, 1H, H(8)), 7.05 (s, 1H, H(11)), 6.81 (s, 1H, H(4)), 5.79 (d, *J* = 1.9 Hz, 1H, =CHC(O)OCH_3_), 5.24 (s, 2H, CH_2_OAc), 3.83 (s, 3H, OCH_3_(1)), 3.76 (s, 3H, =CHC(O)OCH_3_), 3.60 (s, 3H, OCH_3_(2)), 3.37 (s, 3H, OCH_3_(3)), 2.98–2.90 (m, 1H, CH_2_(5)), 2.70–2.61 (m, 1H, CH_2_(5)), 2.48–2.41 (m, 2H, CH_2_(6)), 2.09 (s, 3H, CH_2_OAc).

^13^C NMR (101 MHz, DMSO-*d*_6_, δ, ppm): 169.9, 165.7, 161.6, 153.4, 153.3, 152.3, 150.8, 140.5, 137.7, 135.8, 129.3, 127.6, 124.1, 122.9, 118.7, 110.4, 107.5, 107.0, 60.5, 60.34 57.9, 55.8, 51.0, 38.4, 30.1, 20.6.

Elemental analysis: found (%): C, 66.94; H, 5.62. C_26_H_26_O_8_. Calcd (%): C, 66.97; H, 5.58.

MS (EI): *m*/*z* (%) = 466 (100), 407 (7), 406 (4).

**9Z**: m.p. 144–146 °C.

^1^H NMR (400 MHz, DMSO-*d*_6_, δ, ppm, *J*/Hz): 7.64 (s, 1H, H(12)), 7.33 (s, 1H, H(8)), 7.04 (s, 1H, H(11)), 6.78 (s, 1H, H(4)), 5.88 (d, *J* = 1.8 Hz, 1H, =CHC(O)OCH_3_), 5.24 (s, 2H, CH_2_OAc), 3.81 (s, 3H, OCH_3_(1)), 3.76 (s, 3H, =CHC(O)OCH_3_), 3.36 (s, 3H, OCH_3_(2)), 3.28 (s, 3H, OCH_3_(3)), 3.02 (dd, *J* = 14.8, 6.0 Hz, 1H, CH_2_(6)), 2.83 (td, *J* = 14.5, 5.5 Hz, 1H, CH_2_(6)), 2.60 (dd, *J* = 13.6, 5.1 Hz, 1H, CH_2_(5)), 2.45–2.34 (m, 1H, CH_2_(5)), 2.10 (s, 3H, CH_2_OAc).

^13^C NMR (101 MHz, DMSO-*d*_6_, δ, ppm): 170.0, 164.6, 157.8, 153.2, 152.7, 152.0, 150.9, 140.7, 135.3, 134.6, 128.7, 127.1, 125.3, 122.3, 118.5, 109.5, 108.1, 107.2, 60.6, 60.3, 58.0, 55.8, 50.6, 43.2, 29.7, 20.6.

Elemental analysis: found (%): C, 66.94; H, 5.62. C_26_H_26_O_8_. Calcd (%): C, 66.98; H, 5.64.

MS (EI): *m*/*z* (%) = 466 (100), 407 (13), 406 (2).

## Figures and Tables

**Table 1:** Antiproliferative activity of compounds **8a**–**c**, **9Z** and **9E** (IC_50_, nM ^a^).

Compound	COLO-357	BxPC-3	HaCaT	PANC-1	A549
Colchicine (**1**)	16	16	3	80	400
**8a**	>2000	>2000	>2000	– ^b^	–
**8b**	160	30	32	32	30
**8c**	32	5	32	6	6
**9E**	32	6	0.3	−	–
**9Z**	800	160	300	–	–

^a^ IC_50_ is the concentration at which a 50% inhibition of cell proliferation is achieved after incubation for 72 h; ^b^ Not tested.

## Data Availability

Not applicable.

## Supplementary Materials

The following supporting information can be downloaded at: https://www.mdpi.com/article/10.3390/pharmaceutics15041034/s1, File S1: copies of ^1^H and ^13^C NMR spectra.
