# Supplementary material for: Design, Synthesis and In Vitro Biological Activity of Novel C-7 Methylene Congeners of Furanoallocolchicinoids
Source: Pharmaceutics. 2023 Mar 23;15(4):1034. doi: 10.3390/pharmaceutics15041034 (PMC10143105; doi:10.3390/pharmaceutics15041034)
Supplement: Supplementary file 1 [file pharmaceutics-15-01034-s001.zip › pharmaceutics-2279911-supplementary.pdf]

Supporting information for

Design, Synthesis and In Vitro Biological Activity of Novel C-7 Methylene Congeners of Furanoallocolchicinoids

Iuliia A. Gracheva [1], Elena V. Svirshchevskaya [2], Ekaterina S. Shchegravina [1], Yulia B. Malysheva [1], Alsu R. Sitdikova [1] and Alexey Yu. Fedorov [1,*]

[1] Department of Organic Chemistry, Nizhny Novgorod State University, Gagarina Av. 23, Nizhny Novgorod 603950, Russia

[2] Department of Immunology, Shemyakin-Ovchinnikov Institute of Bioorganic Chemistry RAS, Miklukho-Maklaya 16/10, Moscow 117997, Russia

^{1}H and ^{13}C NMR spectra of compounds 14, 15a-c, 8a-c, 9Z, 9E

Compound 14

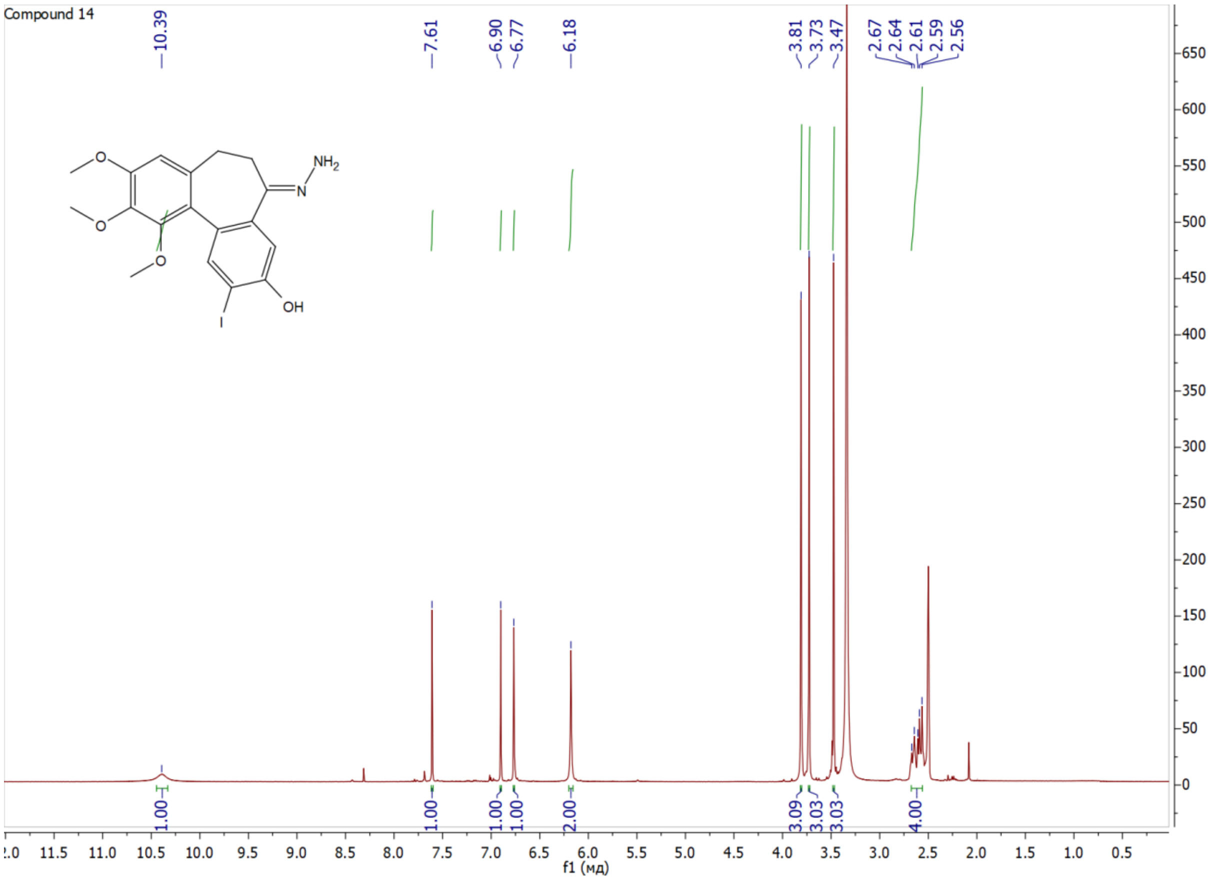

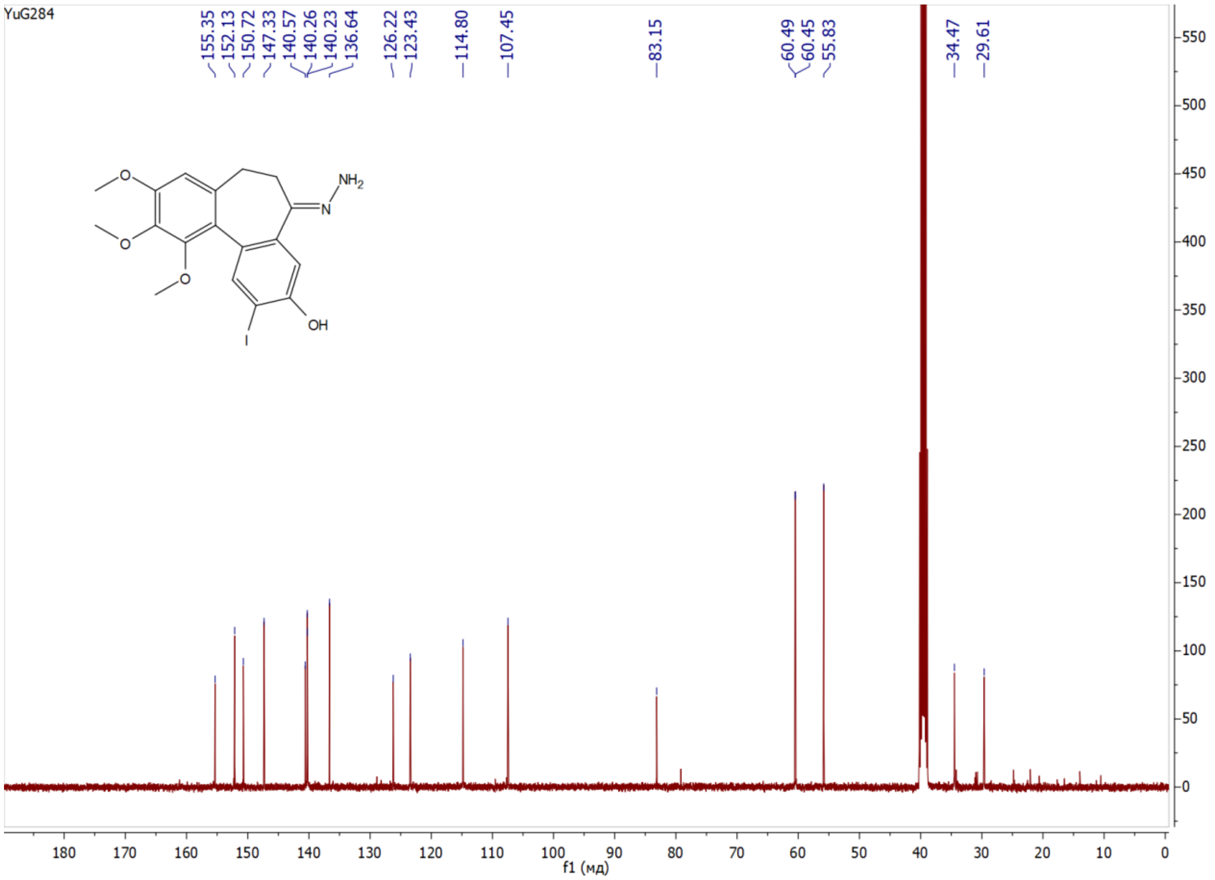

Compound 15a

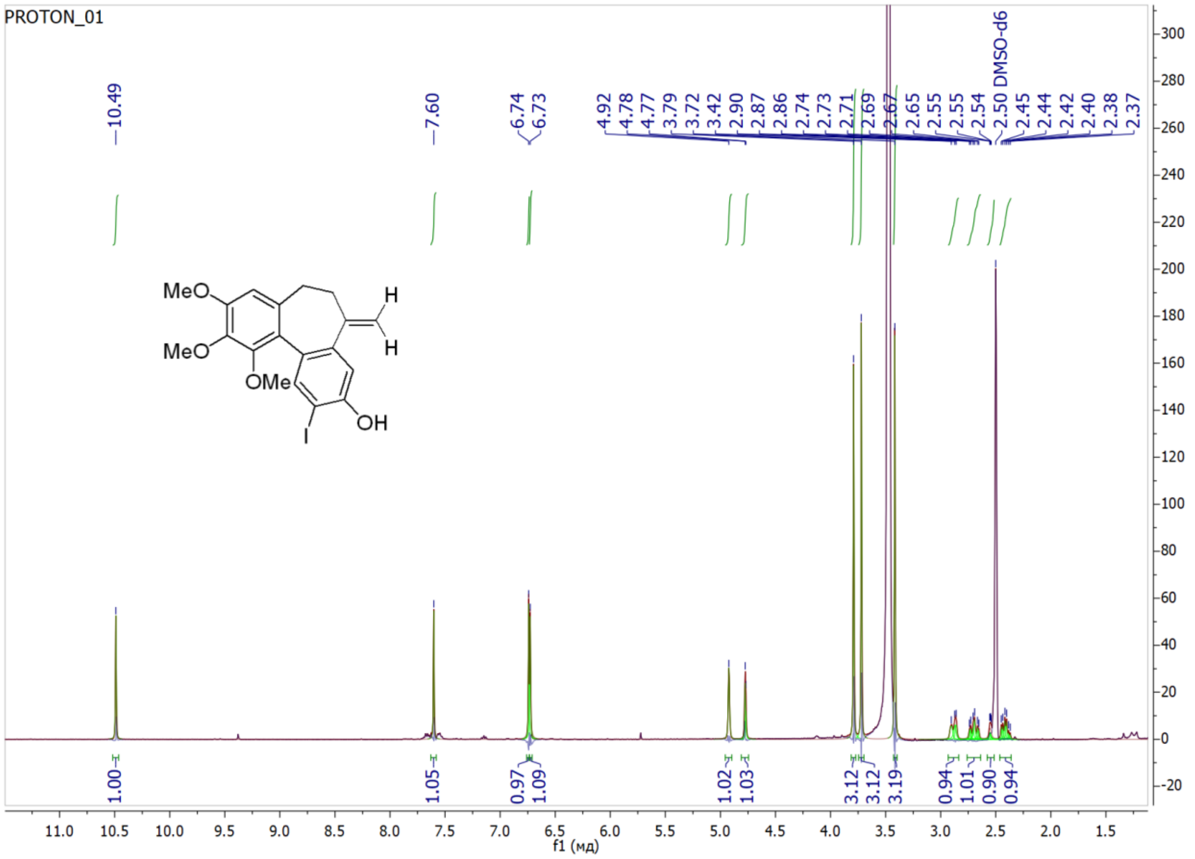

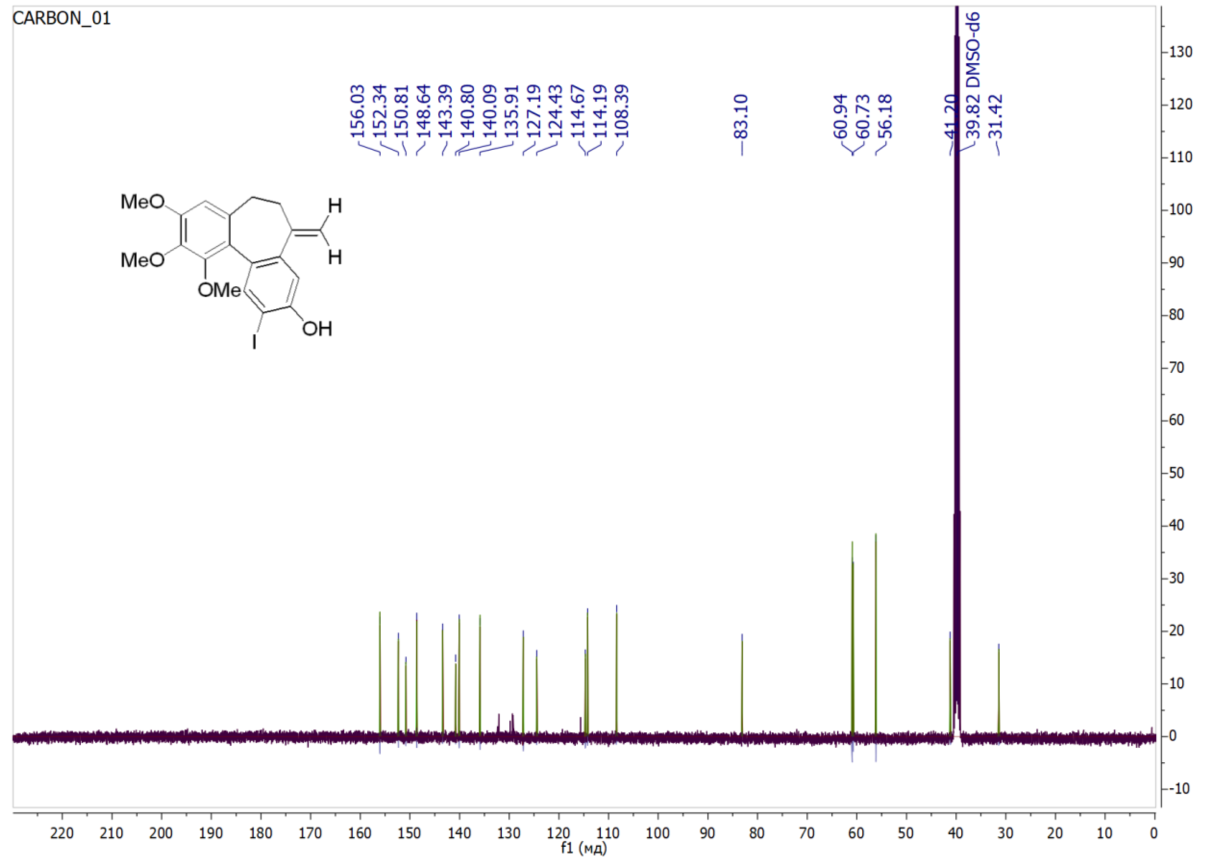

Compound 15b

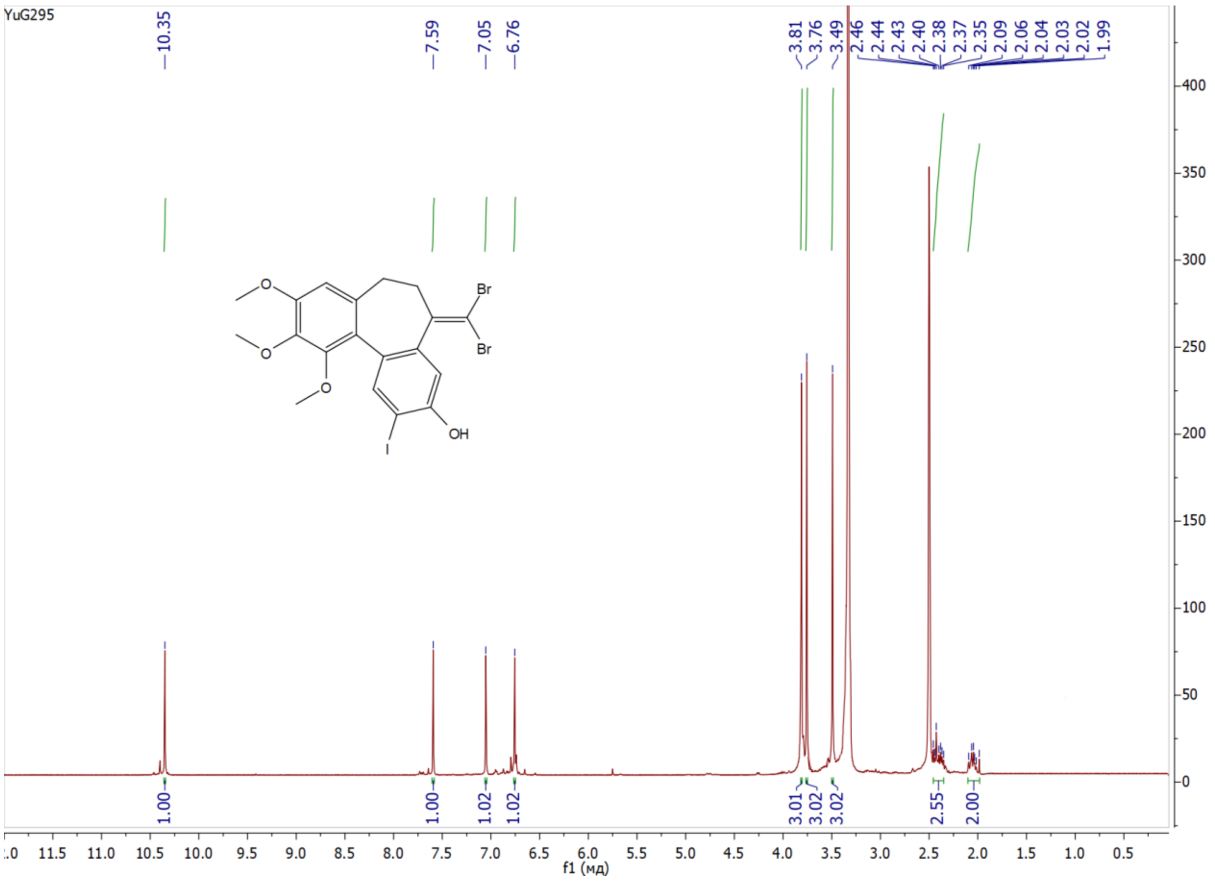

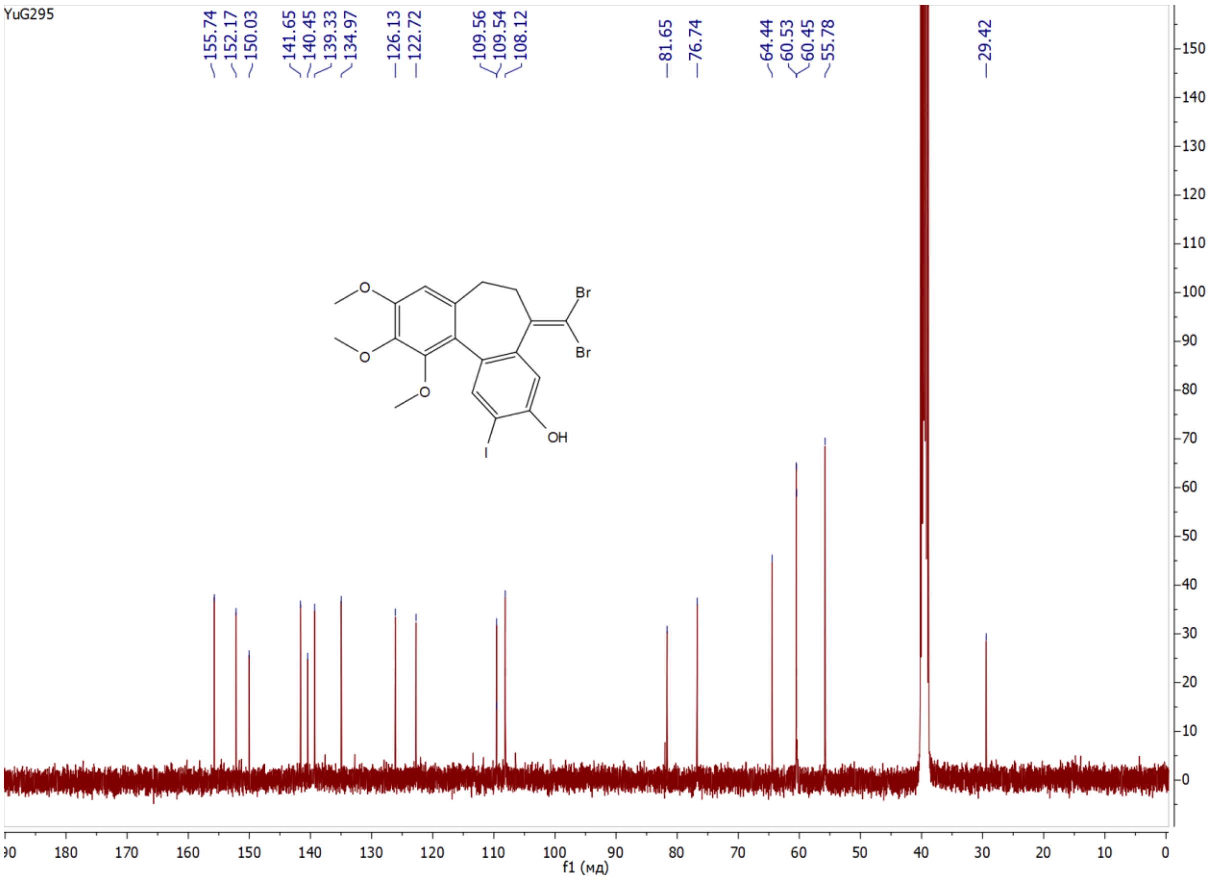

Compound 15c

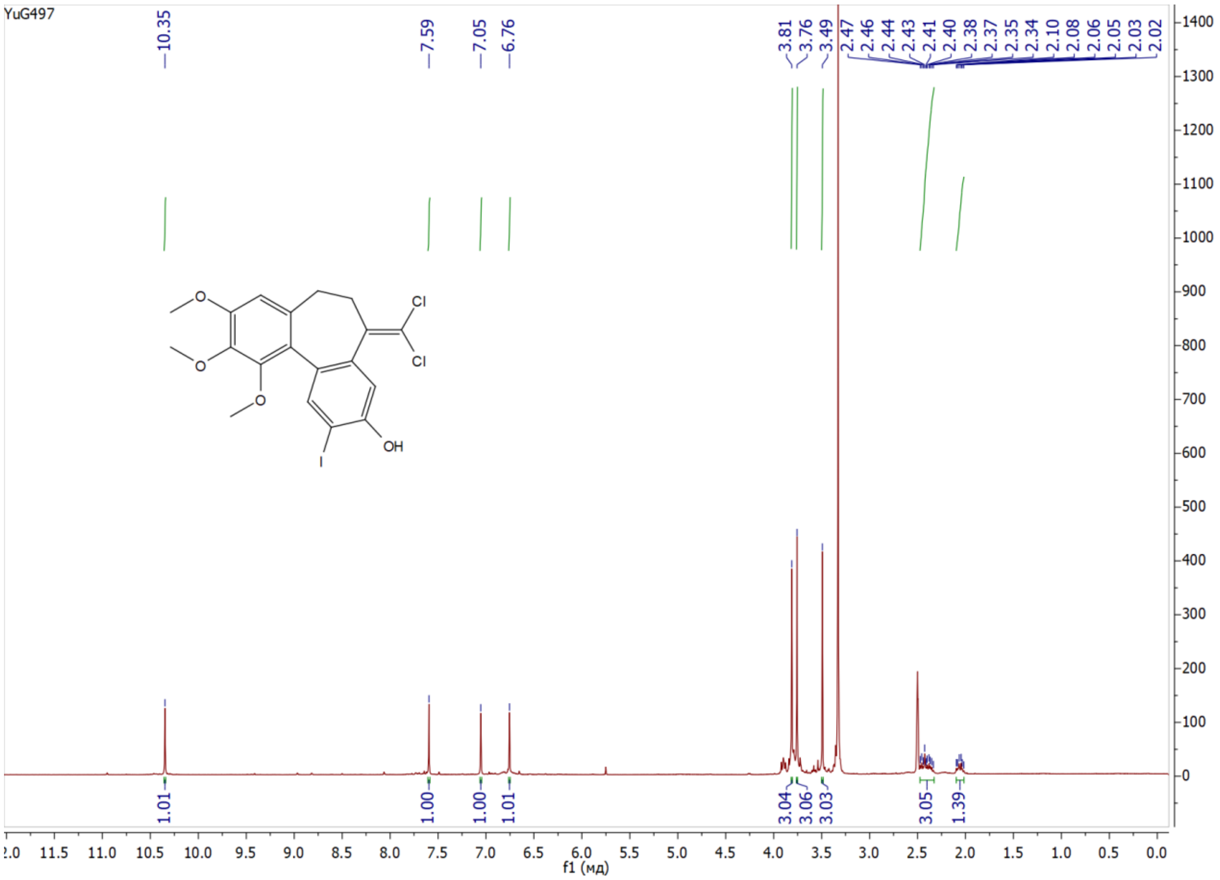

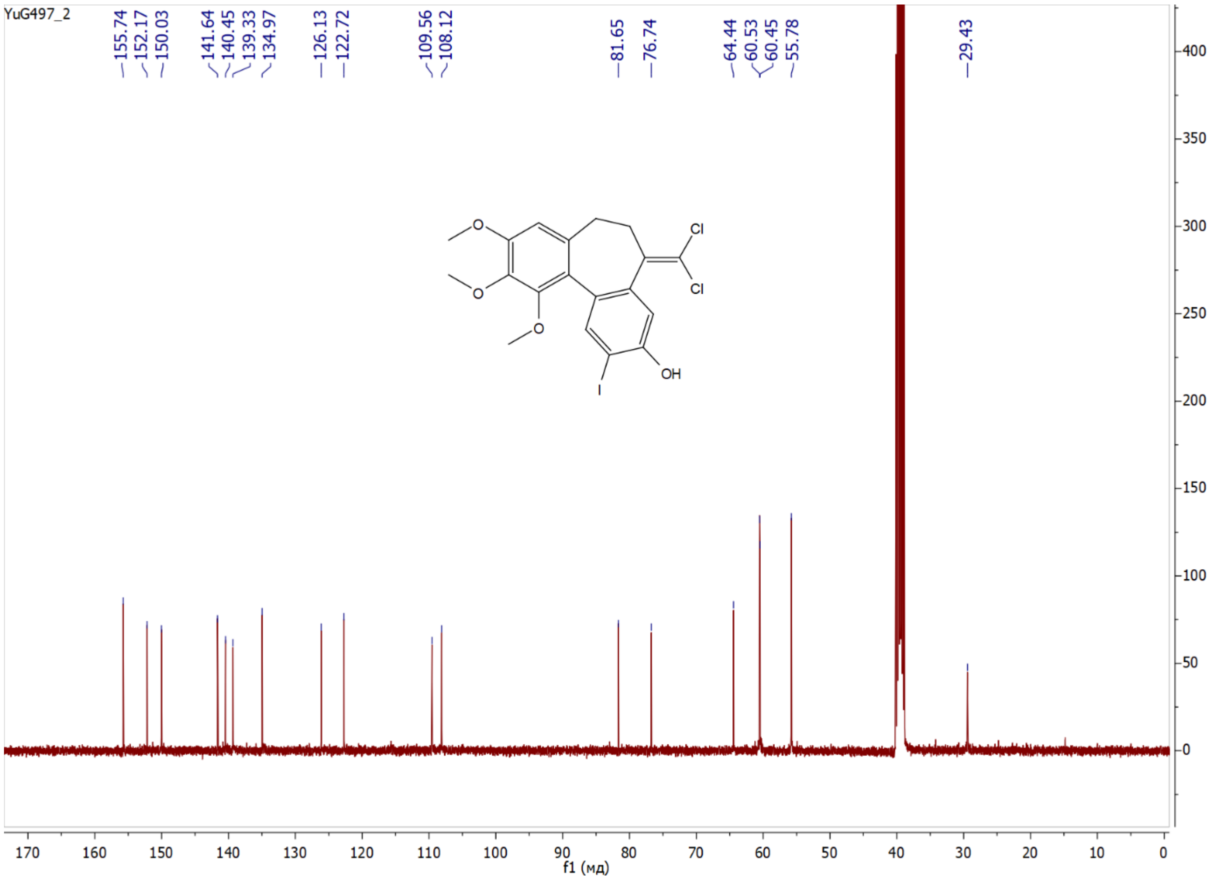

Compound 8a

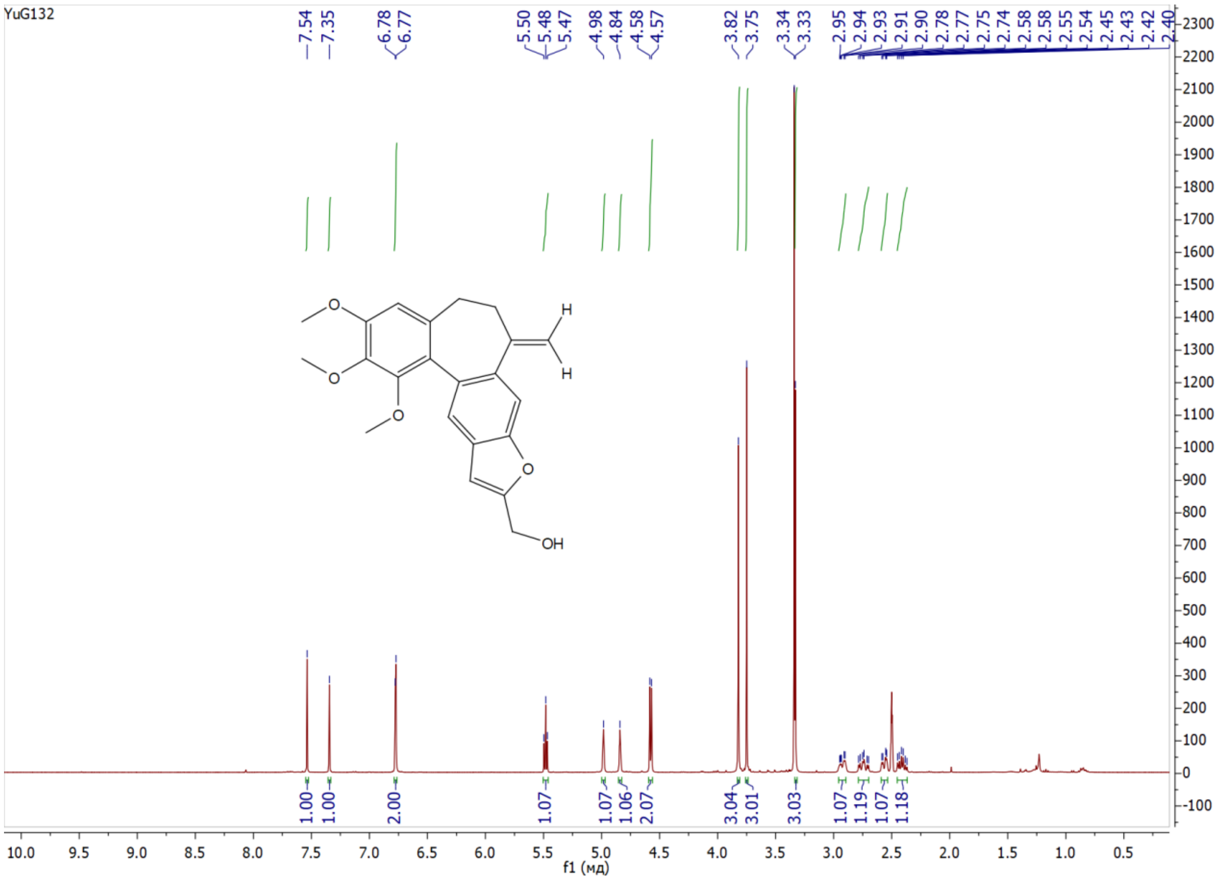

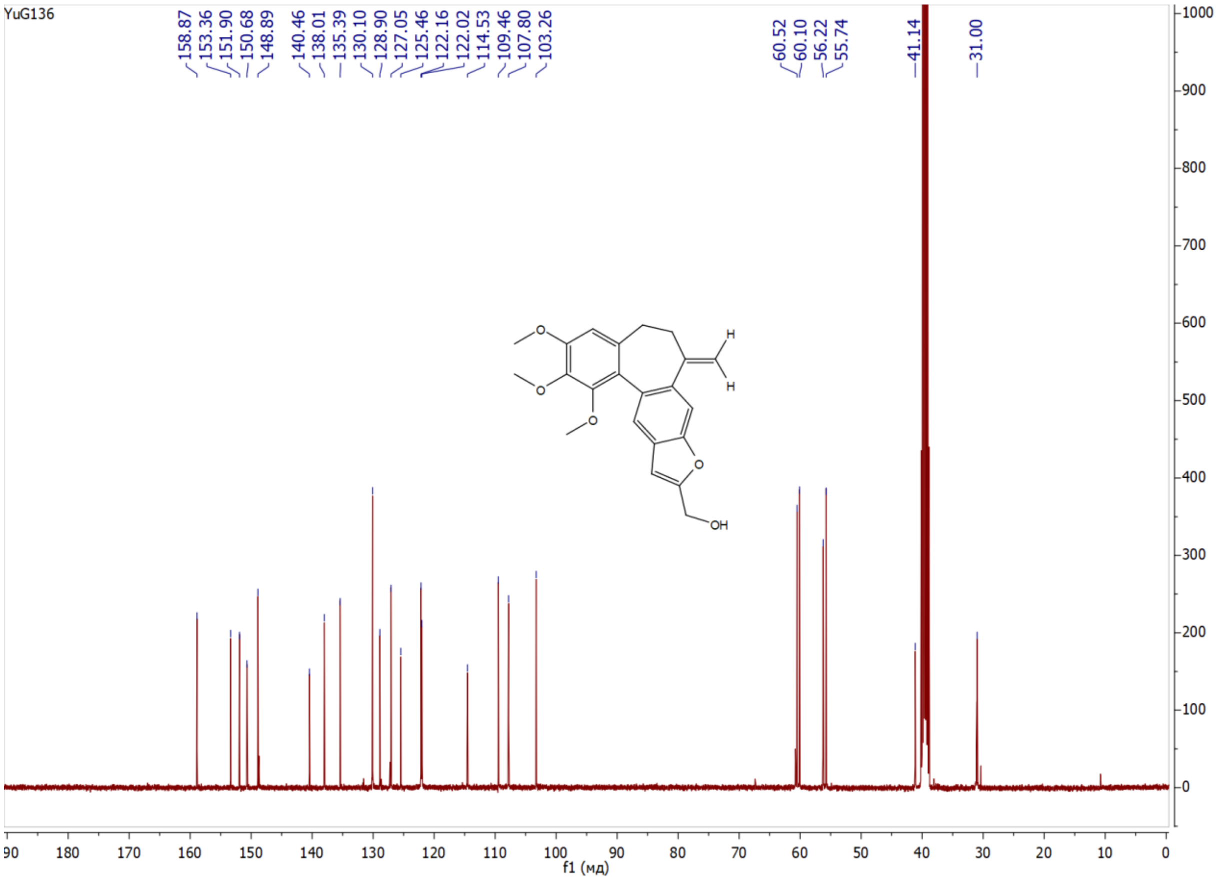
YuG136
158.87
153.36
151.90
150.68
148.89
140.46
138.01
135.39
130.10
128.90
127.05
125.46
122.16
122.02
114.53
109.46
107.80
103.26
60.52
60.10
56.22
55.74
41.14
31.00
H
H
O
O
O
O
OH
f1 (мд)

Compound 8b

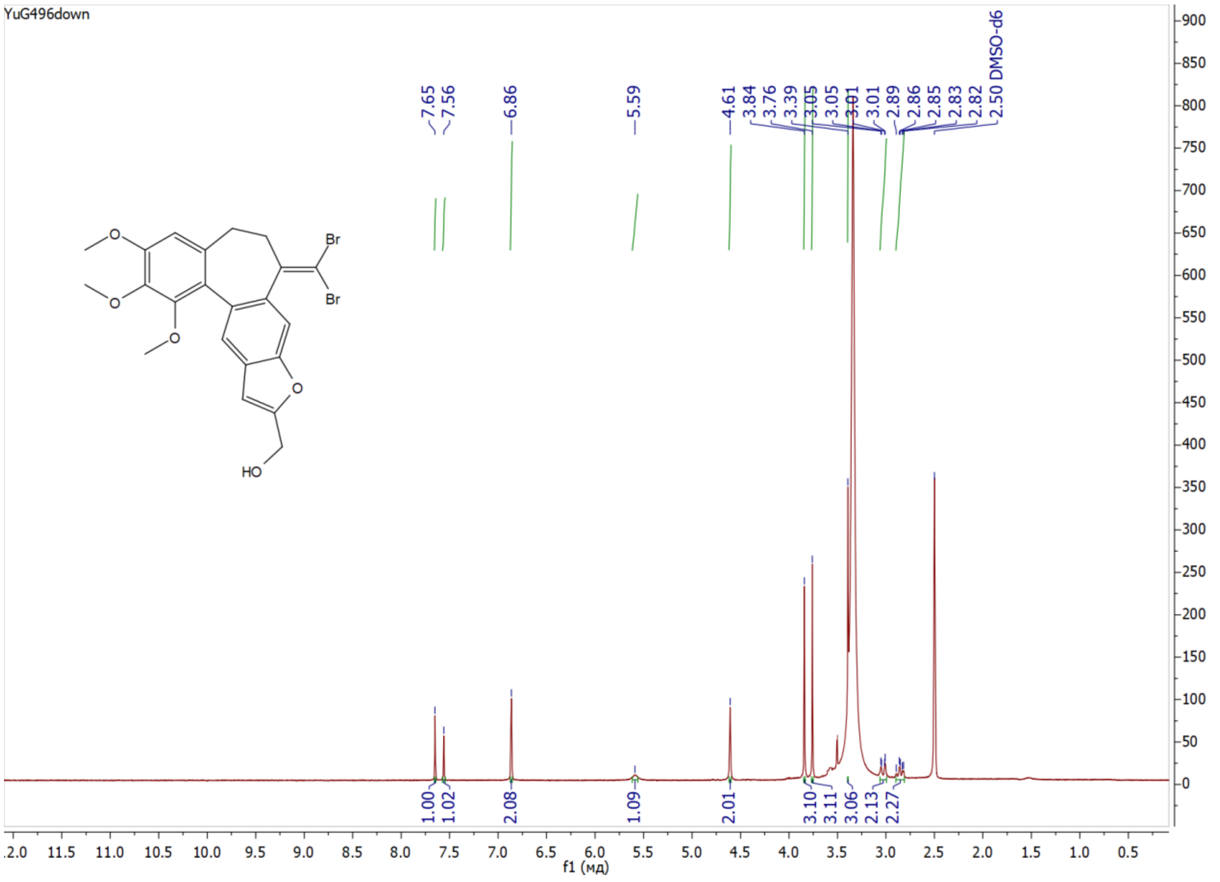
YuG496down
7.65
7.56
6.86
5.59
4.61
3.84
3.76
3.39
3.05
3.01
3.01
2.89
2.86
2.85
2.83
2.82
2.50 DMSO-d6
1.00
1.02
2.08
1.09
2.01
3.10
3.11
3.06
2.13
2.27
Br
Br
O
O
O
O
HO
f1 (мд)

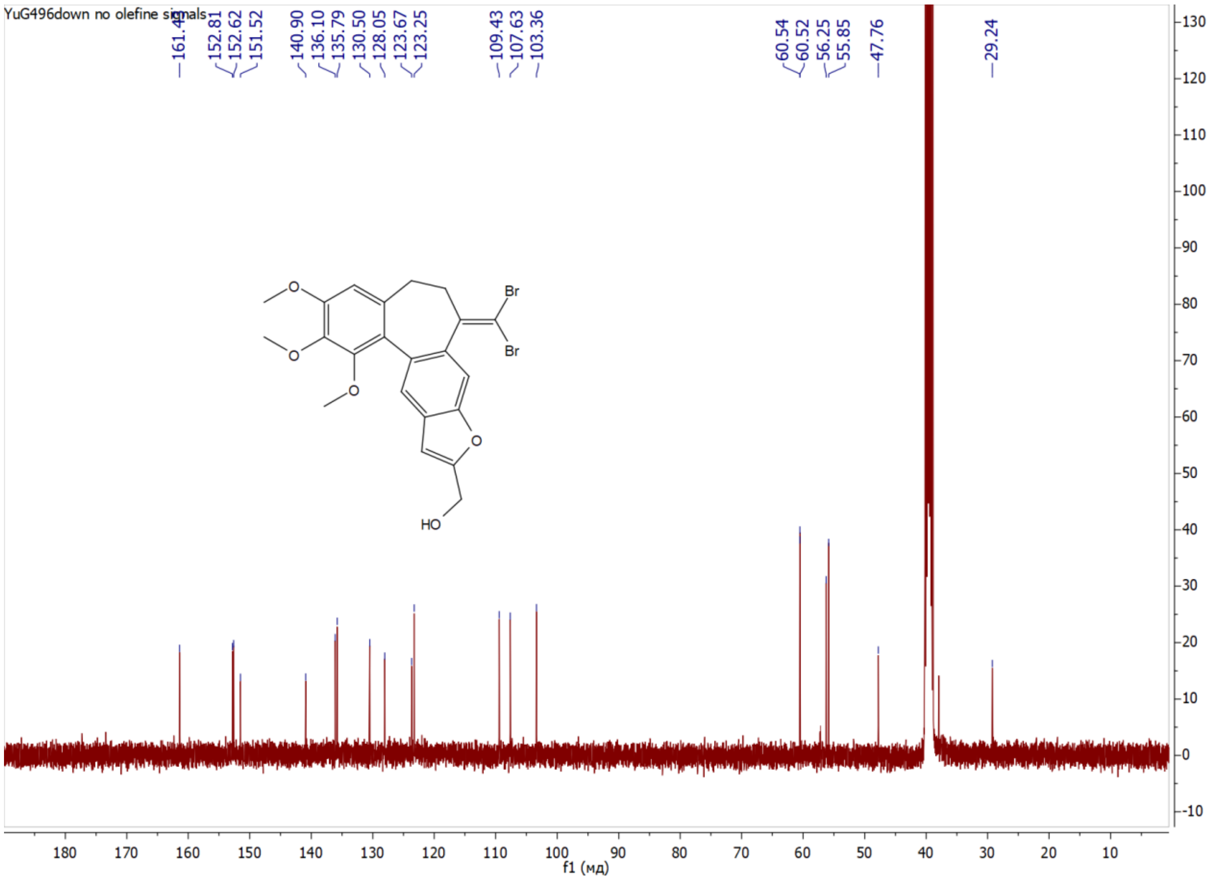
YuG496down no olefine signals
152.81
152.62
151.52
140.90
136.10
135.79
130.50
128.05
123.67
123.25
109.43
107.63
103.36
60.54
60.52
56.25
55.85
47.76
29.24
Br
Br
O
O
O
O
HO
f1 (мд)

Compound 8c

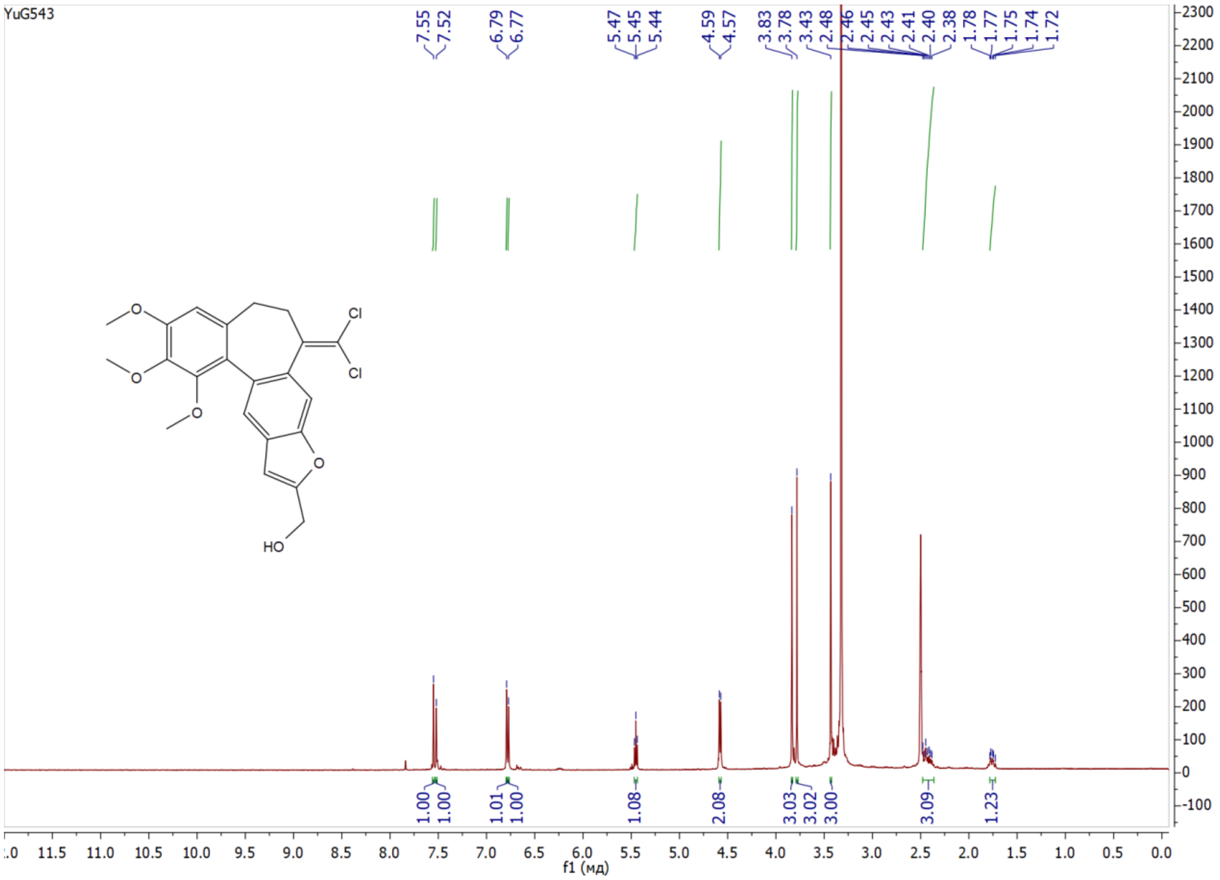
YuG543
7.55
7.52
6.79
6.77
5.47
5.45
5.44
4.59
4.57
3.83
3.78
3.43
2.48
2.46
2.45
2.43
2.41
2.40
2.38
1.78
1.77
1.75
1.74
1.72
Cl
Cl
O
O
O
O
HO
1.00
1.00
1.01
1.00
1.08
2.08
3.03
3.02
3.00
3.09
1.23
f1 (мд)

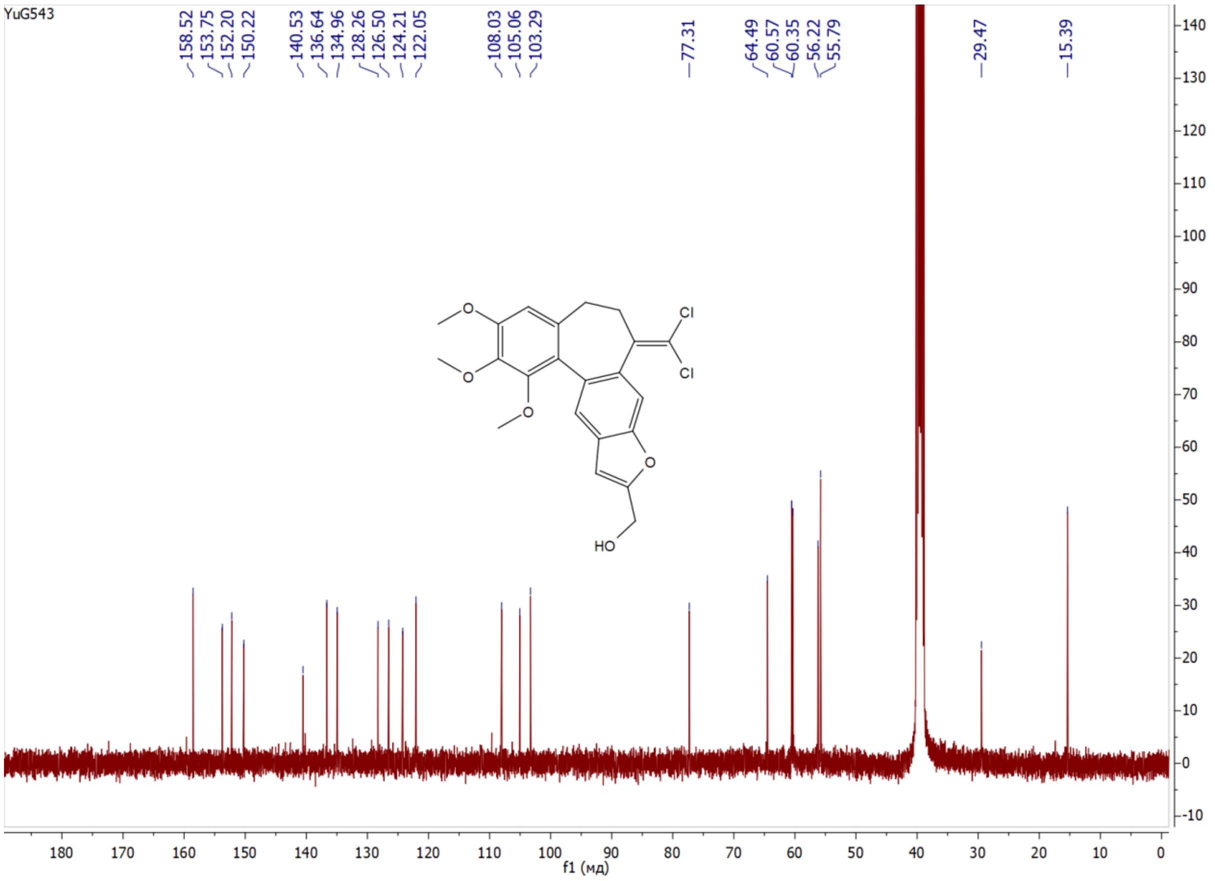
YuG543
158.52
153.75
152.20
150.22
140.53
136.64
134.96
128.26
126.50
124.21
122.05
108.03
105.06
103.29
77.31
64.49
60.57
60.35
56.22
55.79
29.47
15.39
Cl
Cl
O
O
O
O
HO
f1 (мд)

Compound 9Z

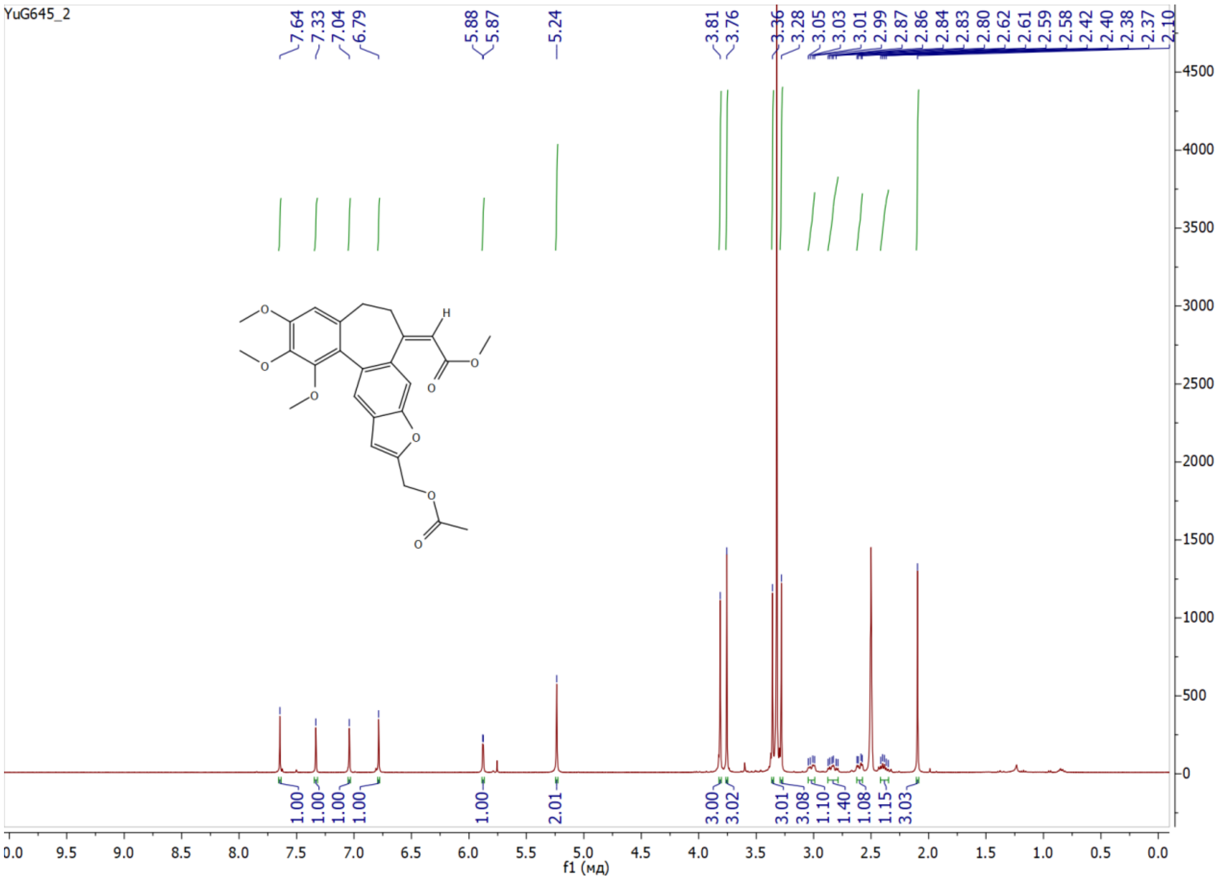
YuG645_2
7.64
7.33
7.04
6.79
5.88
5.87
5.24
3.81
3.76
3.36
3.28
3.05
3.03
3.01
2.99
2.87
2.86
2.84
2.83
2.80
2.62
2.61
2.59
2.58
2.42
2.40
2.38
2.37
2.10
1.00
1.00
1.00
1.00
1.00
2.01
3.00
3.02
3.01
3.08
1.10
1.40
1.08
1.15
3.03
H
O
f1 (мд)

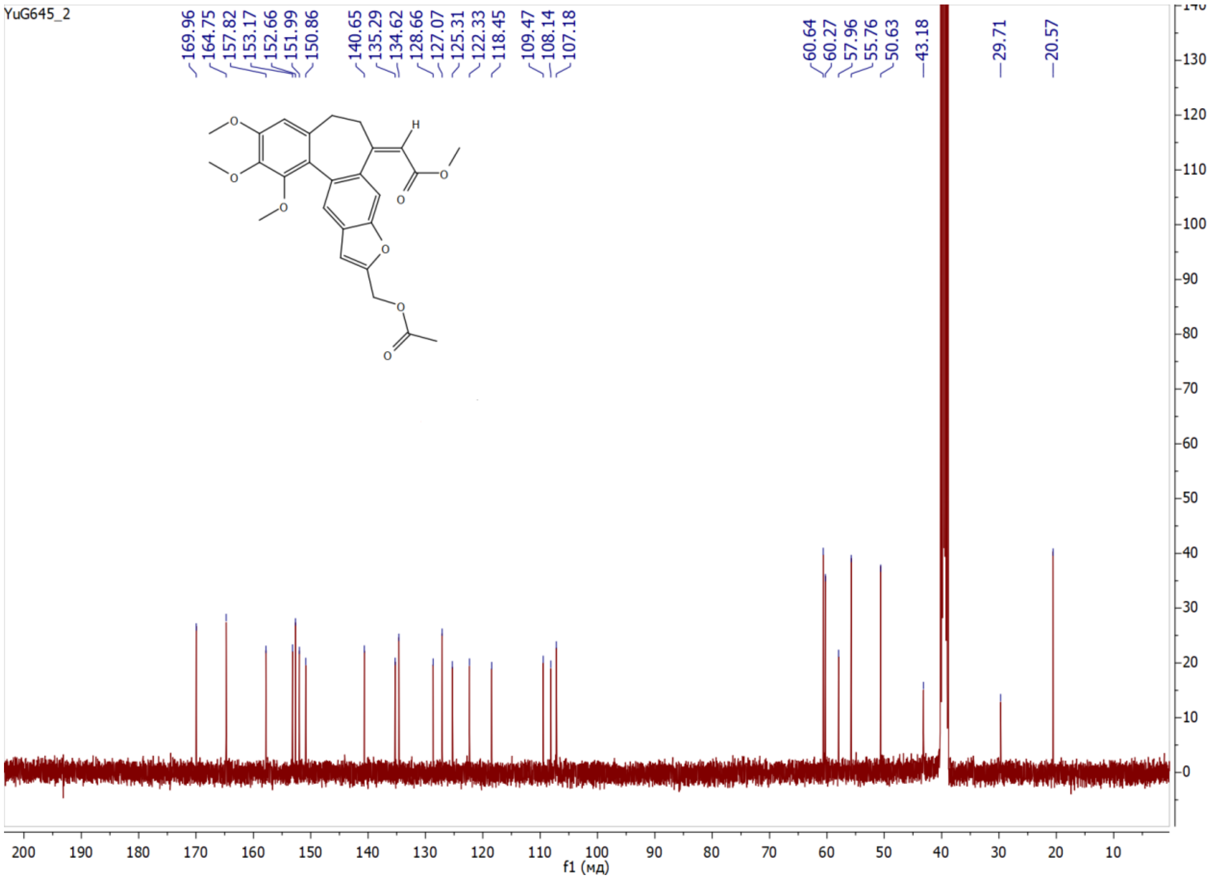
YuG645_2
169.96
164.75
157.82
153.17
152.66
151.99
150.86
140.65
135.29
134.62
128.66
127.07
125.31
122.33
118.45
109.47
108.14
107.18
60.64
60.27
57.96
55.76
50.63
43.18
29.71
20.57
H
O
f1 (мд)

Compound 9E

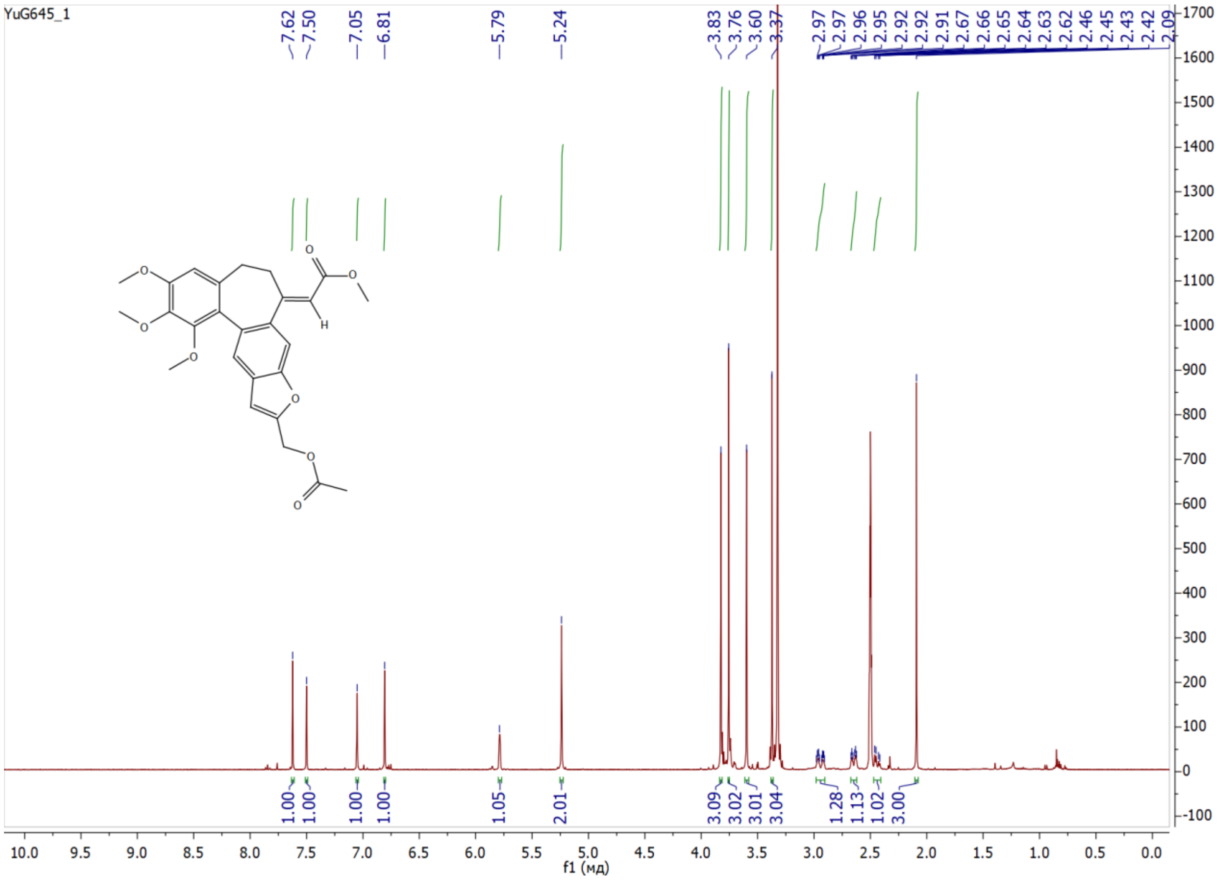
YuG645_1
7.62
7.50
7.05
6.81
5.79
5.24
3.83
3.76
3.60
3.37
2.97
2.97
2.96
2.95
2.92
2.92
2.91
2.67
2.66
2.65
2.64
2.63
2.62
2.46
2.45
2.43
2.42
2.09
1.00
1.00
1.00
1.00
1.05
2.01
3.09
3.02
3.01
3.04
1.28
1.13
1.02
3.00
f1 (мд)

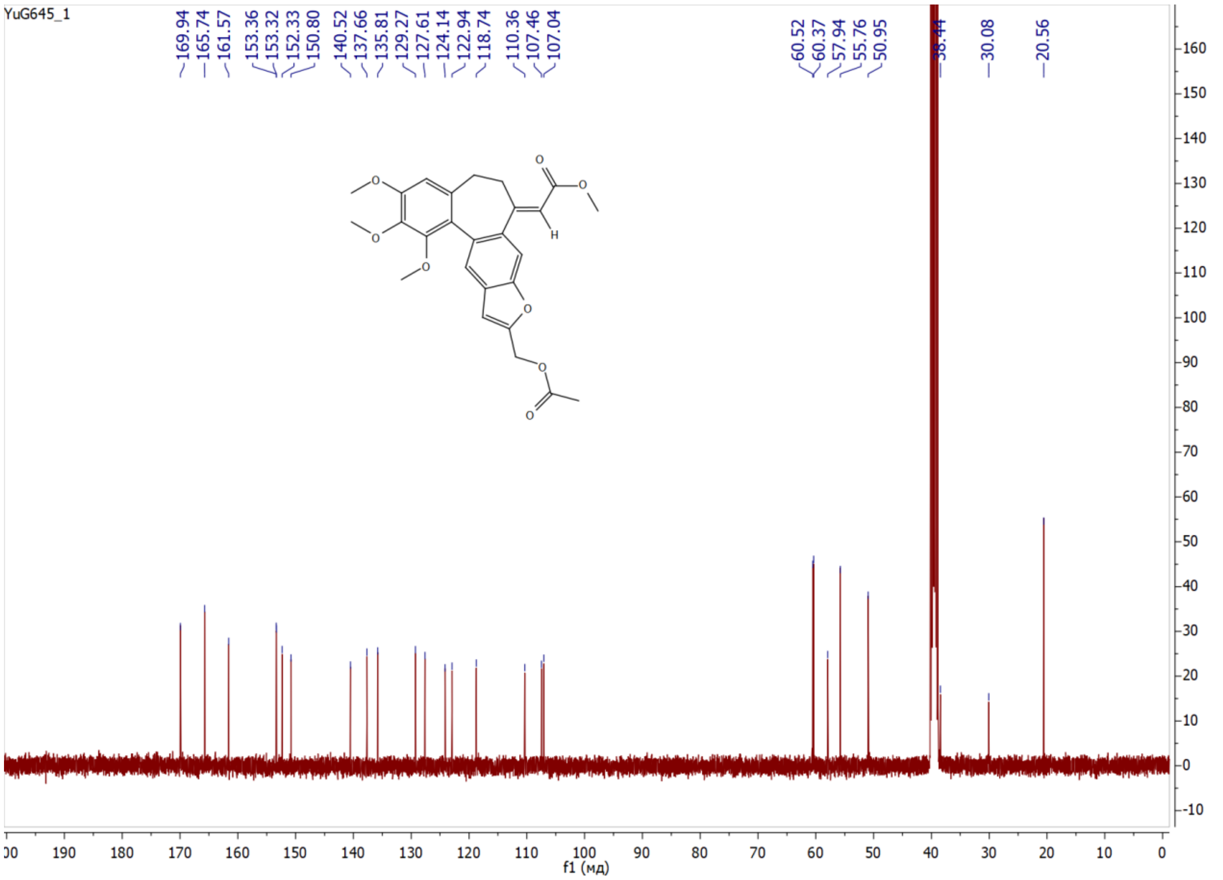
YuG645_1
169.94
165.74
161.57
153.36
153.32
152.33
150.80
140.52
137.66
135.81
129.27
127.61
124.14
122.94
118.74
110.36
107.46
107.04
60.52
60.37
57.94
55.76
50.95
30.08
20.56
f1 (мд)

Explanation of the assignment of *Z*- and *E*-configuration for compounds 9Z and 9E

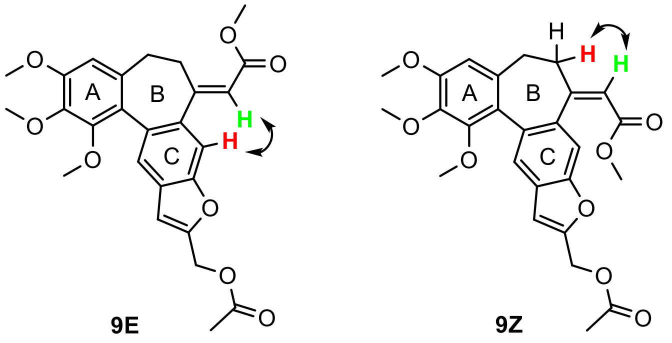

The assignment of E-/Z-configuration of the mentioned isomers (compounds **9E** and **9Z**) were made according to 2D NOESY spectra. The NOESY spectrum of **9E**-isomer showed the through space interaction of olefinic proton (marked in green) with aromatic proton of C-ring (marked in red). In contrast for **9Z**-isomer it was found the interaction of olefinic proton (marked in green) with one of the protons of methylene group of B-ring (marked in red).

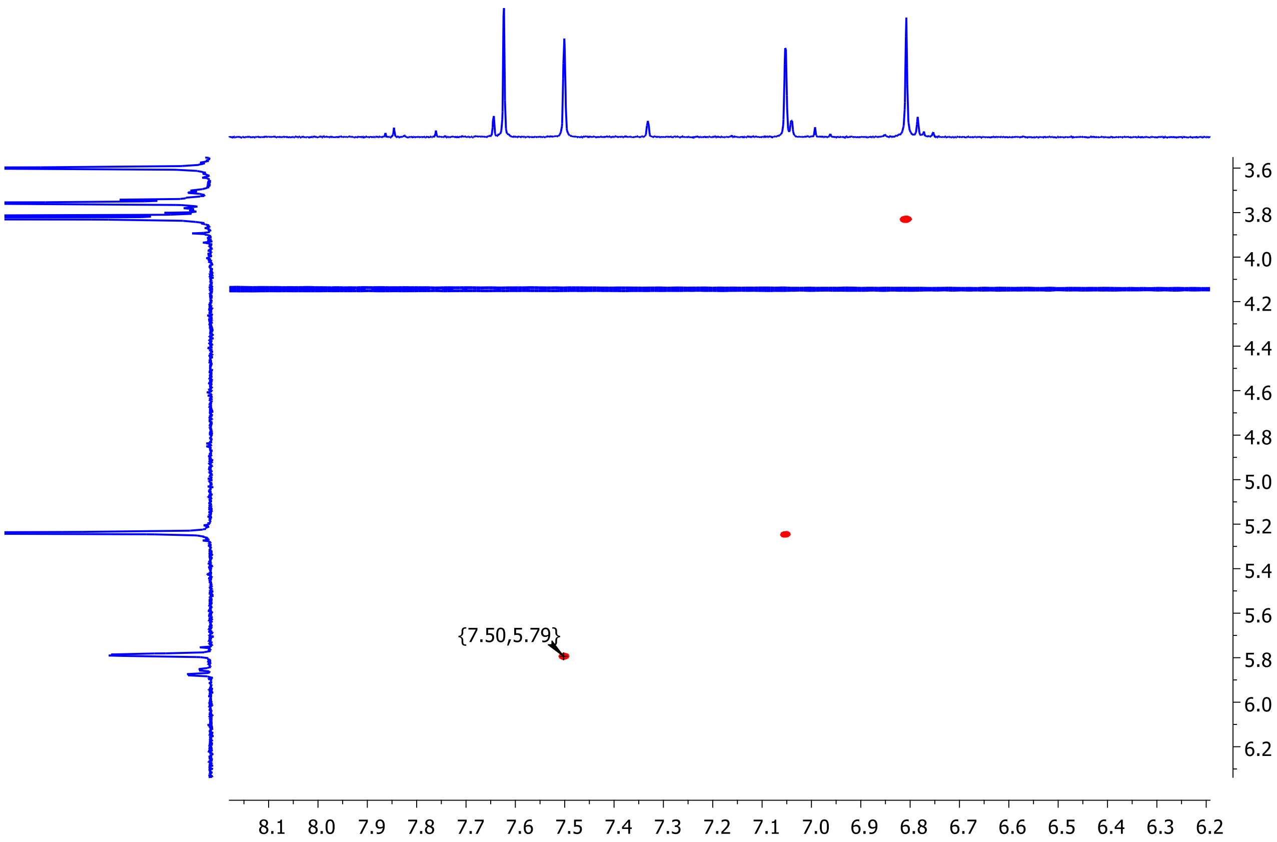

Figure S1. 2D NOESY spectrum of compound **9E**.

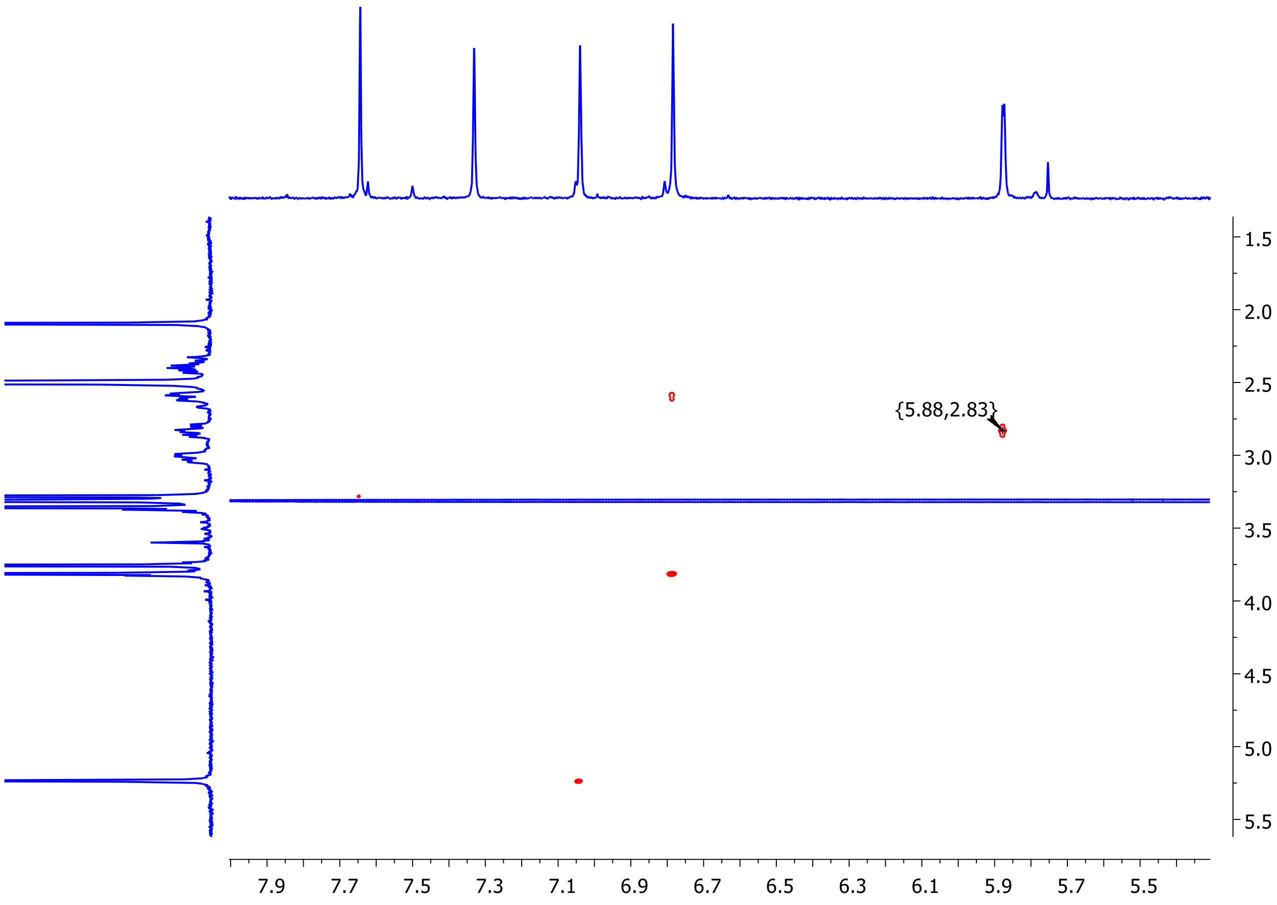

Figure S2. 2D NOESY spectrum of compound **9Z**.
